# Overview of historical formaldehyde occupational exposure in China

**DOI:** 10.1093/annweh/wxaf037

**Published:** 2025-07-15

**Authors:** Jia Nie, Calvin B Ge, Nathaniel Rothman, Wei Hu, Qing Lan, Roel Vermeulen, Susan Peters

**Affiliations:** Institute for Risk Assessment Sciences, Utrecht University, Yalelaan 2, 3584 CM Utrecht, the Netherlands; Netherlands Organization for Applied Scientific Research TNO, Princetonlaan 6, 3584 CB Utrecht, the Netherlands; Division of Cancer Epidemiology and Genetics, National Cancer Institute, 9609 Medical Center Drive, Bethesda, MD 20892, United States; Division of Cancer Epidemiology and Genetics, National Cancer Institute, 9609 Medical Center Drive, Bethesda, MD 20892, United States; Division of Cancer Epidemiology and Genetics, National Cancer Institute, 9609 Medical Center Drive, Bethesda, MD 20892, United States; Institute for Risk Assessment Sciences, Utrecht University, Yalelaan 2, 3584 CM Utrecht, the Netherlands; Institute for Risk Assessment Sciences, Utrecht University, Yalelaan 2, 3584 CM Utrecht, the Netherlands

**Keywords:** occupational exposure, formaldehyde, China

## Abstract

**Objectives:**

Formaldehyde is a known human carcinogen related to leukemia and nasopharyngeal cancer. As China is the world’s largest producer and consumer of formaldehyde, occupational exposure to formaldehyde may pose potential health risks to workers. We aimed to describe occupational exposure to formaldehyde over time in China.

**Methods:**

Occupational formaldehyde exposure measurements were extracted from Chinese and English scientific publications as well as routine occupational hazard monitoring datasets. A weighted mean concentration was calculated by occupation and industry.

**Results:**

We extracted over 20,447 individual measurements from 73 industries and 70 occupations during 1979 to 2023 across China. The majority of measurements (19%) were from the industry “Manufacture of veneer sheets and wood-based panels,” with a pooled mean task-based concentration of 0.69 (0.02 to 4.98) mg/m^3^. Among occupations with over 200 individual measurements and a pooled weighted mean concentration of 0.5 mg/m^3^ or higher, “Metal moulders and coremakers” has the highest task-based concentration, at 1.40 (0.04 to 1.99) mg/m^3^. Formaldehyde exposure levels varied across occupations and changed over time. Before 1990, the overall pooled mean (range) task-based concentration was 1.60 (0.15 to 6.14) mg/m^3^, decreasing to 0.41 (0.00 to 12.0) mg/m^3^ from 2011 onward.

**Conclusions:**

Occupational formaldehyde exposure in China has shown a declining trend over the past decades but remains high in certain occupations. Identifying high-risk industries and occupations can inform the development of targeted interventions and regulations to mitigate formaldehyde exposure. Furthermore, the presented exposure data can contribute to better exposure assessment in epidemiological investigations.

**What’s Important About This Paper?**

Comprising 246 Chinese and English scientific literature and available measurement datasets, this review summarizes formaldehyde exposure in occupational settings in China over time. Over the years, a general downward trend in occupational formaldehyde exposure levels has been found. Several industries and occupations related to chemicals and wood products manufacturing, as well as the use of formaldehyde as a disinfectant and preservative, were identified with high exposure levels.

## Introduction

Formaldehyde (CH_2_O) is a widely used chemical across industries since it was commercially produced in 1889. The International Agency for Research on Cancer (IARC) has classified formaldehyde as a human carcinogen (group 1) (IARC 2006). Acute exposure to formaldehyde can irritate the skin, throat, lungs, and eyes (NIOSH 2014) and numerous studies confirmed the causal relationship between formaldehyde exposure and the risk of leukemia and nasopharyngeal cancer (IARC 2006).

Formaldehyde is used to manufacture wood products, textiles, plastics, chemicals, disinfectants, and preservatives (Cogliano et al. 2004). Three main industries where workers may be exposed to formaldehyde have been identified by IARC: (i) the production of formaldehyde and/or its solutions; (ii) the production or use of products containing formaldehyde; and (iii) combustion processes generating formaldehyde (IARC 2012). With the rapid growth of formaldehyde supply and demand industries, China has led in formaldehyde production and consumption worldwide since the 21st century (Cui 2003). The widespread production and utilization pose significant occupational health challenges (Tang et al. 2009). Thus, understanding formaldehyde exposure patterns in Chinese occupational settings may provide valuable insights for investigating adverse health effects and developing targeted interventions and regulations.

A previous review in 2009 extensively summarized formaldehyde production, consumption, exposure, and health effects in China (Tang et al. 2009). Analyzing 1,265 occupational exposure measurements from five industries and anatomical and pathological laboratories between 1985 and 2006, the review identified generally elevated exposure levels, with the highest concentrations in the wood industry and medical laboratories. Meanwhile, a decreasing trend in exposure levels was observed following the implementation of new occupational exposure limit (OEL) standards. Initially, the Chinese Ministry of Health established an OEL of 3 mg/m³ in 1979 under the *Chinese National Standard for industrial premises (TJ 36-79)*. This limit was reduced to 0.5 mg/m³ in 2002 with the implementation of *the (GBZ1-2002) Hygienic Standards for the Design of Industrial Premise*s, which remains in effect today. However, the previous review, covering a few industries, lacked details on job titles and sampling types (i.e., whether these were area or personal measurements or task-based or full-shift concentrations).

Several types of literature may report formaldehyde occupational exposure levels (National Health Commission of the People’s Republic of China, 2024): (i) Industrial Hygiene Surveys involving systematic measurements conducted in various workplaces (e.g., several factories) to assess potential environmental risks associated with occupational exposures; (ii) Occupational Hazard Assessment comprehensively evaluating all types of risks (including environmental factors, physical, biological, psychological, etc.) at the workplace with a focus on workers’ risk assessment (usually involving the calculation of exposure rating, determination of risk levels, and risk management); (iii) Occupational poisoning case-reports usually conducted when an acute occupational poisoning case has occurred; (iv) Broader environmental air quality studies that include workplace measurements. (v) Epidemiological studies such as case–control studies that conducted measurements at different workplaces to explore the association between occupational exposure and the risk of disease or certain symptoms.

This paper offers an overview of current and historical occupational formaldehyde exposure across all relevant industries in China. Available data from various English and Chinese sources are aggregated, gathering evidence from published resources and individual measurement datasets from routine occupational hazard monitoring.

## Methods

Comprehensive searches of electronic databases were conducted in both the English and Chinese languages. For English, PubMed, Embase, and Web of Science were searched. For Chinese, searches were conducted in the China National Knowledge Infrastructure database, the China Science and Technology Journal Database (VIP), and the WanFang database. Search methods were adjusted according to the different Chinese and English databases. The search included all the indexed papers and computerized literature databases, supplemented by manual screening reference lists from each relevant article.

Furthermore, representative measurements were extracted from a database containing task-based and full-shift personal and area measurements collected between 1985 and 2018 as part of the routine occupational hazard monitoring in Tianjin and Hong Kong. The PRISMA flowchart of data extraction is shown in **Supplementary Figure 1**.

### Search strategy

Search Terms used for the literature review were: All fields= ((Formaldehyde OR formalin OR methanal OR formal OR methylene oxide) AND (China OR Chinese OR Hongkong)) AND (((Occupational AND exposure) OR (industrial AND exposure) OR (work AND exposure) OR (workplace air concentration)) OR ((Occupational hazard) OR (occupational risk) OR (occupational health) OR (industrial hygiene))). The search was done on 3 April 2024. The search strings in Chinese used in different Chinese databases are listed in **Supplementary Material 1**.

### Eligibility criteria

Any publication reporting formaldehyde measurements conducted at workplaces in China was considered. We restricted our inclusion to publications in English or Chinese. We had no restriction on the time period. The most complete report was included when multiple publications were based on the same dataset. Publications or measurements related to acute poisonings, extremely high formaldehyde concentrations that would be immediately dangerous for health, or duplicate publications in different journals were excluded.

### Data extraction and synthesis

The relevant information was extracted from publications or measurement datasets. For each identified set of summary statistics (any form of concentration level including single values, means with or without standard deviations, quantiles, ranges, etc.), we extracted related information including industry, job title and/or job task, sample year, location, area or personal sampling, number of measurements and other available information listed in **Table S1**. All the extracted information was translated, coded, and tabulated. Short-term exposure level (STEL) and time-weighted average (TWA) concentration were defined as task-based and full-shift concentration, respectively; when none specified, we defined the type of concentration based on other information such as sampling duration. The job titles and industries were coded according to the International Standard Industrial Classification of All Economic Activities (ISIC4) (United Nations 2008) and the International Standard Classification of Occupations (ISCO88) (International Labour Organization 2024).

### Analysis

For each extracted set of summary statistics, the arithmetic mean (AM) was calculated for summarization, as it was the predominant form of reported concentration. If a single formaldehyde concentration value was reported, it was considered as an individual measurement. If the authors only provided a range of values, the AM of the minimum and maximum reported values was used as the average. If only a maximum value was provided, the AM was calculated as the average of the maximum and zero. If only the minimum value was given, the observation was considered missing. When only the median and/or geometric mean (GM) were provided, it was used as the AM as only few publications reported these statistics. When “undetected” was reported, the value was set as 0.

Weighted linear regression was conducted to evaluate the temporal trends in exposure levels. The log-transformed task-based AM concentrations (normally distributed) derived from sets of summary statistics were used as the dependent variable, and years of measurement served as the independent variable. We weighted the analysis by the number of measurements for each set of summary statistics. This analysis could not be extended to full-shift concentrations due to the limited availability of summary statistics.

We pooled the AM concentrations of each job title weighted by the number of measurements reported for the summary statistics. This was calculated by multiplying the number of sites measured by the number of samples taken per site. In circumstances where only one of these metrics was provided (either the number of sites measured or the number of samples taken per site), it was assumed that at least that many measurements were taken. In circumstances where none of the abovementioned sampling information was given, we assigned “at least 1” for a single concentration value and “at least 2” for a range of concentration or a mean value with standard deviation (SD).

## Results

After excluding publications without assessments specific to an industry or occupation, 246 publications (**Supplementary Material 2**) were included in the analysis. These publications comprised 826 sets of summary statistics and over 20,131 individual measurements, covering the period from 1979 to 2023. Most of the 826 sets of summary statistics reported AMs with SDs or ranges (33%), single values (27%, could be either the concentration of one measurement, or an AM without SDs or ranges), or ranges only (33%), while only 6% reported Medians and/or GMs. Among all sets of summary statistics, 11% had only one measurement. Further, 316 representative measurements were extracted from Tianjin’s occupational hazard monitoring (*n* = 313) and Hong Kong (*n* = 3). In total, we pooled 20,447 individual assessments from 73 industries and 70 occupations; most reports provided task-based formaldehyde air concentrations (**Table 1**, **Tables S2**  **and**  **Supplementary S3**).

**Table 1.** Pooled mean formaldehyde concentrations (mg/m^3^) by occupation in China, 1979-2023.

Job Title (ISCO88)	Task-based^*^	No. measurements	Full-shift^*^	No. measurements
Life science and health professionals (2200)	0.26 (0.14–1.79)	49		
Pharmacologists, pathologists, and related professionals (2212)	0.92 (0.02–12.0)	512	0.51 (0.51–0.51)	2
Librarians and related information professionals (2432)	0.07 (0.00–0.08)	230		
Office clerks (4100)	0.06 (0.02–0.27)	1098	0.08 (0.07–0.40)	42
Stock clerks (4131)	0.63 (0.05–1.62)	100		
Personal and protective services workers (5100)	0.08 (0.07–0.13)	50		
Travel attendants and travel stewards (5111)	0.01 (0.00–0.07)	833		
Waiters, waitresses, and bartenders (5123)	0.06 (0.01–0.09)	87		
Hairdressers, barbers, beauticians, and related workers (5141)	0.05 (0.02–0.10)	1383		
Stall and market salespersons (5230)	0.12 (0.01–1.11)	1659		
Stone splitters, cutters, and carvers (7113)	0.74 (0.72–1.10)	25		
Building finishers and related trade workers not elsewhere classified (7130)	0.30 (0.29–1.27)	1094		
Plumbers and pipe fitters (7136)	0.03 (0.03–0.03)	896		
Painters and related workers (7141)	1.12 (1.12–1.12)	602		
Varnishers and related painters (7142)	0.32 (0.00–1.70)	40	0.33 (0.07–4.70)	174
Metal moulders and coremakers (7211)	1.40 (0.04–1.99)	368	0.30 (0.07–0.56)	23
Upholsterers and related workers (7437)	0.17 (0.02–0.32)	36		
Well drillers and borers and related workers (8113)	3.75 (0.00–5.80)	47		
Glass and ceramics kiln and related machine operators (8131)	1.37 (0.04–2.31)	32		
Wood-processing-plant operators (8141)	0.68 (0.02–4.98)	3569	0.62 (0.09–1.48)	20
Chemical-processing-plant operators (8150)	0.50 (0.01–5.00)	434	1.55 (0.98–1.57)	405
Crushing-, grinding- and chemical-mixing-machinery (8151)	0.19 (0.03–0.52)	43	0.38 (0.07–1.87)	19
Still and reactor operators (except petroleum and natural gas) (8154)	2.09 (0.00–9.10)	36	7.84 (0.15–8.80)	10
Incinerator, water treatment, and related plant operators (8163)	0.95 (0.00–1.56)	852		
Pharmaceutical-and toiletry-products machine operators (8221)	0.21 (0.01–2.70)	233		
Metal finishing-, plating-, and coating-machine operators (8223)	0.02 (0.02–0.02)	72		
Rubber-products machine operators (8231)	0.46 (0.02–2.00)	120		
Plastic-products machine operators (8232)	0.72 (0.02–9.90)	257	0.94 (0.13–8.70)	42
Wood-products machine operators (8240)	0.33 (0.05–3.80)	560	0.07 (0.07–0.07)	3
Printing-machine operators (8251)	0.15 (0.03-0.56)	69		
Fibre-preparing, spinning- and winding-machine operators (8261)	0.95 (0.22–5.59)	69		
Bleaching-, dyeing- and cleaning-machine operators (8264)	0.09 (0.03–0.10)	124		
Food and related products machine operators (8270)	0.09 (0.06–0.12)	144		
Brewers, wine, and other beverage machine operators (8278)	0.06 (0.06–0.06)	30		
Mechanical-machinery assemblers (8281)	2.68 (0.17–-8.30)	13	0.24 (0.03–0.56)	386
Electronic-equipment assemblers (8283)	0.26 (0.00–1.30)	289		
Metal-, rubber- and plastic-products assemblers (8284)	0.18 (0.18–0.18)	96		
Wood and related products assemblers (8285)	0.74 (0.03–3.33)	41	0.18 (0.12–0.32)	5
Other machine operators and assemblers (8290)	0.70 (0.01–4.50)	30	0.23 (0.07–1.20)	8
Helpers and cleaners in offices, hotels, and other establishments (9132)	0.06 (0.03–0.09)	1537		
Hand packers and other manufacturing labourers (9322)	0.43 (0.07–0.75)	49		

Notes: Only job titles with at least 25 measurements were listed in the table.

^*^Weighted mean concentrations (minimum-maximum) were calculated for all measurements from both publications and representative measurements, and the minimum and maximum of the reported concentrations were shown in brackets.

Two-thirds of the occupations’ pooled weighted mean formaldehyde exposure concentrations were below the current Chinese national standard of 0.5 mg/m^3^. Occupations with over 200 individual assessments and a pooled weighted mean task-based or full-shift concentration of 0.5 mg/m^3^ or higher included: “Wood-processing-plant operators,” “Incinerator, water-treatment and related plant operators,” “Chemical-processing-plant operators,” “Painters and related workers,” “Pharmacologists, pathologists and related professionals,” “Mechanical-machinery assemblers,” “Metal moulders and coremakers,” and “Plastic-products machine operators.” The occupation with the most exposure measurements was “Wood processing plant operators” (18% of all measurements), with a pooled weighted mean full-shift concentration of 0.68 mg/m³ (range: 0.02 to 4.98 mg/m³) and a task-based concentration of 0.62 mg/m³ (range: 0.09 to 1.48 mg/m³). Among industries, “Manufacture of veneer sheets and wood-based panels,” “Building completion and finishing,” “Sewerage,” “Other human health activities,” “Manufacture of basic iron and steel,” “Manufacture of basic chemicals,” “Manufacture of plastics and synthetic rubber in primary forms,” had more than 200 individual assessments and a pooled weighted mean task-based or full-shift concentration over 0.5 mg/m^3^.

### Time trends of formaldehyde workplace air concentrations

**Figure 1.**
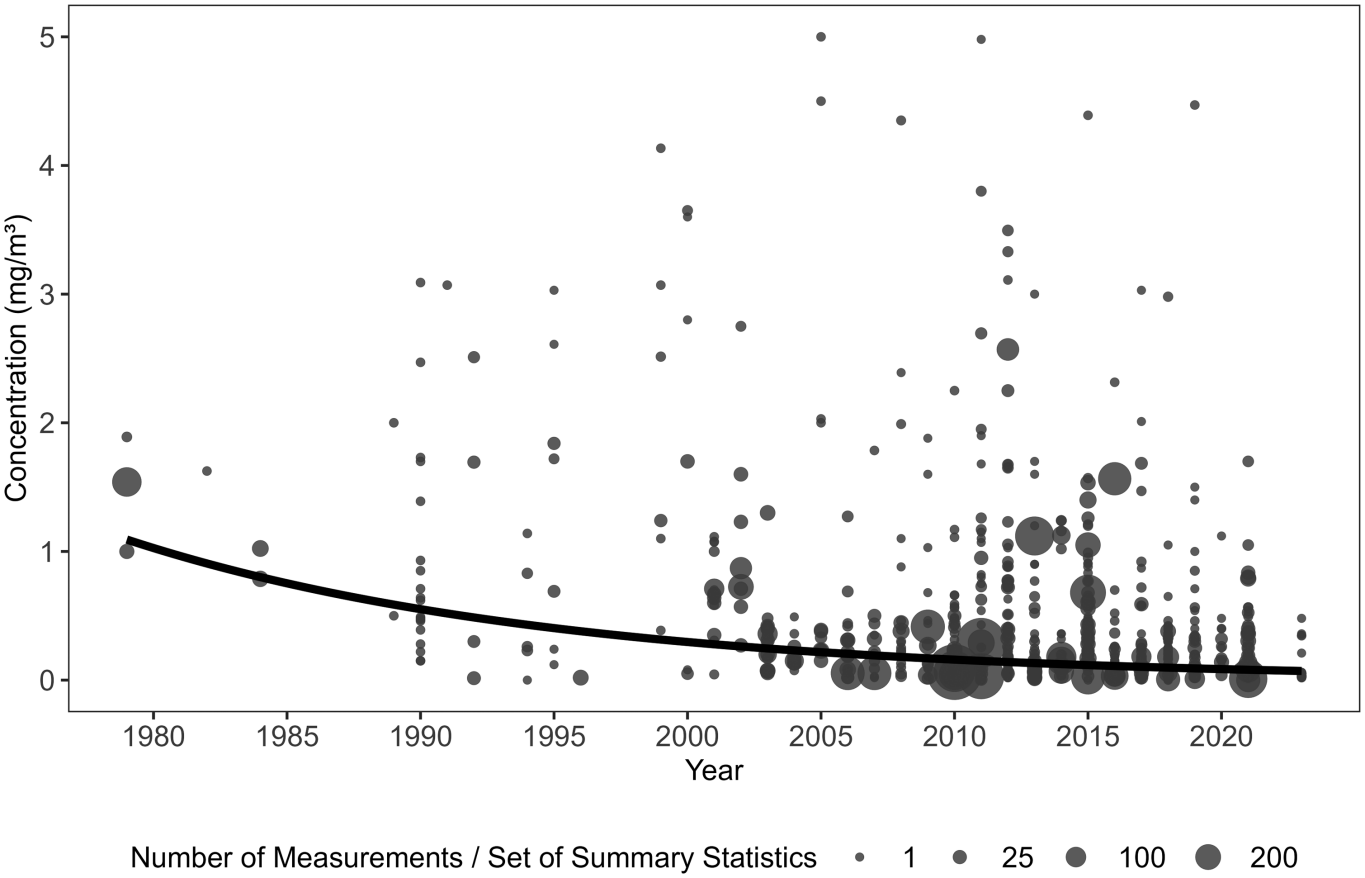
Occupational formaldehyde exposure trends in China, 1979–2023. Notes: Each point represents a task-based arithmetic mean (mg/m^3^) derived from a set of summary statistics, with larger points indicating estimates based on a greater number of measurements. The black line represents the fitted values using weighted linear regression, using log-transformed task-based AMs as the dependent variable and year of measurement as the independent variable. Concentrations exceeding 5.0 mg/m^3^ are omitted from the plot for clarity but are retained in the regression model.

**Figure 1** shows the overall trend of occupational formaldehyde exposure in China from 1979 to 2023. Each point represents a task-based AM (mg/m^3^) derived from a set of summary statistics, with larger points indicating estimates based on a greater number of measurements. The black line represents the fitted weighted linear regression, using log-transformed task-based AMs as the dependent variable and year of measurement as the independent variable. The analysis confirms a significant decreasing trend in exposure levels over time (β = −0.06, *P* < 0.001).

The pattern of formaldehyde exposure per occupation was examined across four time periods: (i) 1990 and earlier; (ii) 1991 to 2000; (iii) 2001 to 2010; and (iv) 2011 and later. Descriptive statistics for these periods are provided in **Table 2** for all occupations collectively and for 3-digit ISCO-88 grouped occupations with over 25 measurements with at least measurements in two time periods. Most measurements were taken after 2000. For all occupations, earlier periods exhibited the highest occupational exposure levels. The pooled weighted mean (range) task-based formaldehyde concentrations in workplaces were 1.60 (0.15 to 6.14) mg/m³ until 1990 and 1.12 (0.00 to 12.8) mg/m³ for 1991 to 2000. The concentrations decreased to 0.24 (0.01 to 9.10) mg/m³ during 2001 to 2010, and then slightly increased to 0.41 (0.00 to 12.0) mg/m³ from 2011 onwards. A similar trend was observed for “Wood-processing and papermaking-plant operators,” though the earliest report for this occupation was from 1991 to 2000. For “Life Science Professionals,” which were mostly pathologists, exposure levels were relatively high during 1991 to 2000, with a pooled weighted mean (range) task-based concentration of 1.50 (0.12 to 3.03) mg/m³. This group experienced a marginal increase in exposure to 1.66 (0.29 to 4.35) mg/m³ during 2001 to 2010, with levels remaining high at 0.90 (0.02 to 12.00) mg/m³ from 2011 onwards. Other occupations with pooled weighted mean concentration over 0.5 mg/m^3^ in the latest decades were “Painters,” “Metal moulders, welders, sheet-metal workers,” “Power production plant operators,” and “Rubber and plastic products machine operators.”

**Table 2.** Pooled mean formaldehyde concentrations (mg/m^3^) grouped by 3-digit ISCO-88 job titles of different historical periods in China.

Job Title (3-digit ISCO-88)	Year period	Task-based^*^	No. measurements	Full-shift^*^	No. measurements
All	1990 and earlier	1.60 (0.15–6.14)	500	4.48 (0.15–8.70)	9
	1991–2000	1.12 (0.00–12.8)	267	0.24 (0.01–0.98)	99
	2001–2010	0.24 (0.00–9.10)	6688	0.79 (0.03–8.80)	994
	2011 and after	0.41 (0.00-12.0)	11314	0.23 (0.03–0.67)	576
Life Science Professionals (221)	Overall	0.92 (0.02–-12.0)	512	0.51 (0.51–0.51)	2
	2011 and after	0.90 (0.02–12.0)	497		
	2001–2010	1.66 (0.29–4.35)	11		
	1991–2000	1.50 (0.12–3.03)	4	0.51 (0.51–0.51)	2
Archivists, Librarians and Related Information Professionals (243)	Overall	0.07 (0.00–0.08)	230		
	2011 and after	0.07 (0.00–0.08)	216		
	1991–2000	0.05 (0.05–0.05)	14		
Material-recording and Transport Clerks (413)	Overall	0.63 (0.05–1.62)	100		
	2011 and after	0.60 (0.05–1.23)	98		
	1990 and earlier	1.62 (1.62–1.62)	2		
Travel attendants and related Workers (511)	Overall	0.01 (0.00–0.07)	833		
	2011 and after	0.01 (0.00–0.02)	802		
	2001–2010	0.06 (0.01–0.07)	31		
Housekeeping and restaurant services workers (512)	Overall	0.06 (0.01–0.09)	87		
	2011 and after	0.07 (0.01–0.09)	60		
	2001–2010	0.06 (0.06–0.06)	27		
Other personal services Workers (514)	Overall	0.05 (0.02–0.10)	1383		
	2011 and after	0.04 (0.02–0.10)	690		
	2001–2010	0.07 (0.07–0.07)	693		
Stall and market Salespersons (523)	Overall	0.12 (0.01–1.11)	1659		
	2011 and after	0.14 (0.07–0.22)	1016		
	2001–2010	0.10 (0.01–1.11)	643		
Miners, shotfirers, stone cutters and carvers (711)	Overall	0.74 (0.72–1.10)	25		
	2011 and after	0.72 (0.72–0.72)	24		
	2001–2010	1.10 (1.10–1.10)	1		
Building finishers and related trades workers (713)	Overall	0.18 (0.03–1.27)	1990		
	2011 and after	0.17 (0.03–0.29)	1980		
	2001–2010	1.27 (1.27–1.27)	10		
Painters, building structure cleaners and related trades workers (714)	Overall	1.07 (0.00–1.70)	642	0.33 (0.07–4.70)	174
	2011 and after	1.08 (0.00–1.70)	632	0.15 (0.07–0.50)	89
	2001–2010	0.69 (0.69–0.69)	10	0.52 (0.07–4.70)	85
Metal moulders,welders, sheet-metalworkers, structural-metal preparers and related trades workers (721)	Overall	1.40 (0.04–1.99)	368	0.30 (0.07–0.56)	23
	2011 and after	0.48 (0.04–0.59)	36	0.30 (0.07–0.56)	23
	2001–2010	1.99 (1.99–1.99)	3		
	1990 and earlier	1.49 (1.00–1.89)	329		
Mining- and mineral-processing-plant operators (811)	Overall	3.75 (0.00–5.80)	47		
	2011 and after	0.06 (0.00–0.08)	15		
	2001–2010	3.25 (0.70–5.80)	4		
	1990 and earlier	5.80 (5.80–5.80)	28		
Wood-processing- and papermaking-plant operators (814)	Overall	0.68 (0.02–4.98)	3569	0.62 (0.09–1.48)	20
	2011 and after	0.83 (0.02–4.98)	1743		
	2001–2010	0.47 (0.05–2.75)	1751	0.62 (0.09–1.48)	20
	1991–2000	1.85 (1.24–3.65)	75		
Chemical-processing-plant operators (815)	Overall	0.59 (0.00–9.10)	516	1.67 (0.07–8.80)	442
	2011 and after	0.17 (0.01–0.90)	309	0.20 (0.07–0.64)	17
	2001–2010	1.25 (0.00–9.10)	81	1.75 (1.57–8.80)	405
	1991–2000	2.02 (1.69–2.51)	30	0.98 (0.98–0.98)	18
	1990 and earlier	0.91 (0.79–1.02)	96	3.98 (0.15–7.80)	2
Power-production and related plant operators (816)	Overall	0.95 (0.00–1.56)	852		
	2011 and after	1.49 (0.00–1.56)	420		
	2001–2010	0.41 (0.41–0.41)	432		
Chemical-products machine operators (822)	Overall	0.17 (0.01–2.70)	305		
	2011 and after	0.17 (0.01–2.70)	304		
	2001–2010	0.25 (0.25–0.25)	1		
Rubber- and plastic-products machine operators (823)	Overall	0.63 (0.02–9.90)	377	0.94 (0.13–8.70)	42
	2011 and after	0.66 (0.02–9.90)	291	0.23 (0.13–0.37)	36
	2001–2010	0.02 (0.02–0.03)	19		
	1991–2000	0.70 (0.23–1.72)	49		
	1990 and earlier	0.68 (0.15–2.00)	18	5.19 (1.05–8.70)	6
Wood-products machine operators (824)	Overall	0.33 (0.05–3.80)	560	0.07 (0.07–0.07)	3
	2011 and after	0.33 (0.05–3.80)	496	0.07 (0.07–0.07)	3
	2001–2010	0.34 (0.08–0.38)	64		
Textile-, fur- and leather-products machine operators (826)	Overall	0.42 (0.03–6.14)	196		
	2011 and after	0.21 (0.03–3.03)	167		
	1991–2000	1.84 (0.08–3.60)	2		
	1990 and earlier	1.65 (0.22–6.14)	27		
Assemblers (828)	Overall	0.36 (0.00–8.30)	439	0.24 (0.03–0.56)	391
	2011 and after	0.27 (0.00–8.30)	368	0.24 (0.03–0.56)	375
	2001–2010	0.84 (0.22–1.30)	71	0.24 (0.07–0.45)	16
Other machine operators and assemblers (829)	Overall	0.70 (0.01–4.50)	30	0.23 (0.07–1.20)	8
	2011 and after	0.28 (0.01–0.77)	25	0.09 (0.07–0.19)	7
	2001–2010	2.78 (0.88–4.50)	5		
	1990 and earlier		0	1.20 (1.20–1.20)	1
Motor-vehicle drivers (832)	Overall	0.09 (0.02–0.52)	33		
	2011 and after	0.10 (0.03–0.52)	31		
	2001–2010	0.03 (0.02–0.03)	2		
Domestic and related helpers, cleaners, and launderers (913)	Overall	0.06 (0.03–2.80)	1539		
	2011 and after	0.07 (0.03–0.91)	125		
	2001–2010	0.06 (0.06–0.06)	1413		
	1991–2000	2.80 (2.80–2.80)	1		

Notes: Only 3-digit job codes with at least 25 measurements and 2 different year period records were listed in the table.

^*^Weighted mean concentrations (minimum-maximum) were calculated for all measurements from both publications and representative measurements, and the minimum and maximum of the reported concentrations were shown in brackets.

### General characteristics

Four main types of studies reported occupational formaldehyde exposure, including case-control studies (21%), industrial hygiene surveys (28%), occupational hazard assessment studies (15%), and environmental air quality inspections in workplaces (34%) (**Table 3**). Among these, occupational hazard assessments reported the highest pooled task-based concentrations, with a weighted mean (range) of 0.87 mg/m³ (0.00 to 12.8 mg/m³). In contrast, concentrations reported from air quality inspections were lower, with a weighted mean (range) of 0.07 mg/m³ (0.00 to 4.35 mg/m³).

**Table 3.** Pooled mean formaldehyde concentrations (mg/m^3^) by data sources in China, 1979-2023.

Data source	Task-based^*^	No. measurements	Full-shift^*^	No. measurements
**Literature review**				
Case–control studies	0.31 (0.01–6.20)	3369	0.71 (0.01–1.57)	903
Industrial hygiene surveys	0.57 (0.00–12.0)	5380	0.58 (0.03–8.80)	393
Occupational hazard assessments	0.87 (0.00–12.8)	3034	0.34 (0.12–0.60)	20
Environmental air quality inspections at the workplace	0.07 (0.00–4.35)	6943	0.26 (0.07–1.48)	62
Other studies	0.35 (0.06–2.00)	27		
**Representative measurements**				
Routine occupational hazard monitoring datasets	0.54 (0.00–6.14)	16	0.29 (0.07–4.70)	300

^*^Weighted mean concentrations (minimum-maximum) were calculated for all measurements from both publications and representative measurements, and the minimum and maximum of the reported concentrations were shown in brackets.

The collected data covered more than 26 provinces, municipalities, and autonomous regions, representing 76% of the 34 administrative regions in China. A subgroup analysis of exposure levels across different regions is shown in **Table S4**. Coastal regions such as Jiangsu, Shandong, Guangdong, and Zhejiang provinces had the most individual exposure measurements. The pooled weighted mean (range) task-based concentrations in these regions were 0.14 (0.02 to 5.59), 0.26 (0.00 to 3.60), 0.76 (0.00 to 4.98), and 0.75 (0.02 to 5.47) mg/m^3^, respectively. Higher exposure levels were found in less developed areas such as the Gansu, Hunan, Sichuan, and Shanxi provinces. The pooled weighted mean (range) task-based concentrations were 3.35 (0.00 to 12.8), 1.44 (0.02 to 9.10), 1.87 (0.02 to 6.20), and 1.24 (0.01 to 8.30) mg/m^3^, respectively.

Most measurements were obtained through area sampling (**Table S5**). Among personal measurements, full-shift concentrations were the most commonly calculated, largely influenced by a case–control study (Zhang et al. 2010) that contributed 846 measurements.

Some source reports, especially those extracted from publications, lacked details on the number of measurements and workplace environmental factors that could influence formaldehyde concentrations and exposures, such as ventilation and other protective procedures. Information on the accuracy and precision of the sampling and analytical methods was often missing; instead, references were frequently made to Chinese Standards. Detailed information on the sampling and analytical methods used in China is provided in **Supplementary Material 3**.

## Discussion

Based on 246 Chinese and English publications and measurements from routine occupational hazard monitoring, our analysis of over 20,447 individual assessments reveals a declining trend of occupational exposure to formaldehyde in China over the past decades. Occupations with a pooled weighted mean task-based air concentration of 0.5 mg/m^3^ or higher typically involved either frequent use or production of formaldehyde, particularly in industries manufacturing chemicals and wood products, and scenarios using formaldehyde as disinfectants and preservatives.

Compared to global assessments spanning 2004 to 2019, which categorized occupational formaldehyde exposure into four main scenarios: healthcare and research, esthetic and wellness, industry, and firefighters (Cammalleri et al. 2022), our review within China revealed a similar pattern but with broader sources of exposure. The primary source of exposure information originated from industries related to the production or use of formaldehyde. Moreover, a secondary set of exposure data was obtained from workplace environmental air quality inspections, particularly in sectors such as short-term accommodation (e.g. hotels) and hairdressing and beauty treatments. A small portion of the data came from the healthcare and research sectors, specifically the workplaces of pathologists and related professionals.

A previous review on formaldehyde exposure in China before 2006 reported that the exposure levels in anatomy and pathology laboratories were high, often exceeding the OEL of 0.5 mg/m^3^, primarily due to the evaporation of formalin used for tissue and specimen preservation (Tang et al. 2009). Our review indicated that the exposure levels among pathologists and related professionals remained high in subsequent time periods, underscoring the need for special attention to ensure a safer working environment.

Most measurements were derived from the coastal and their neighbouring regions, including the Jiangsu, Shandong, Guangdong, Zhejiang, Hubei, and Henan provinces. These regions produced and consumed a large portion of formaldehyde within China, as the long-distance transportation of such a highly reactive chemical is impractical (Tian and Wang 2018). Conversely, less developed inland regions with more rural areas, such as Gansu, Hunan, and Sichuan, exhibited higher levels of exposure in our review. The relatively high exposure levels in these regions were driven by the over-representation of high-exposure occupations. When further analysing the reported industries of developed and less developed areas, we observed that formaldehyde exposure measurements were primarily extracted in manufacturing settings in developed regions, while in inland areas like Sichuan and Gansu, measurements mostly originated from laboratories, including occupations like pathologists.

Although the overall exposure level in occupational settings has decreased over time, especially following the newly issued OEL in 2002, the pooled weighted mean task-based formaldehyde air concentration at workplaces slightly increased in the last decade. Despite the more stringent OEL of 0.5 mg/m^3^ for formaldehyde set by the National Health Commission of the People’s Republic of China, compliance has been inconsistent across different regions and industries (Liang et al. 2003). The expansion of small-scale industries and informal workshops alongside China’s industrialization has contributed to elevated exposure risks, particularly in rural areas where enforcement of the OEL may be less effective (Wong 2003). Moreover, it is important to note that international exposure standards vary: according to the US Occupational Safety and Health Administration (OSHA), the recommended permissible exposure limit (PEL) of formaldehyde is 0.92 mg/m^3^. The recommended exposure limit (REL) of the National Institute for Occupational Safety and Health (NIOSH) for formaldehyde is 0.20 mg/m^3^ (8-hour TWA) (NIOSH 2014). The Threshold Limit Values (TLV) set by the American Conference of Governmental Industrial Hygienists (ACGIH) are 0.12 mg/m^3^ (TWA) and 0.37 mg/m^3^ (STEL) (ACGIH 2016).

Task-based measurements are typically targeted toward the highest exposures and thus expected to be higher than full-shift concentrations. However, this expectation did not hold for all occupations in the current review. This situation was, for instance, seen for “Chemical-processing-plant operators” and “Plastic-products machine operators.”

For the current review, we included information on formaldehyde occupational exposure over an extended period in China, offering a broad view of related occupations and industries. Our overview further integrates data from scientific publications with available measurements from routine occupational hazard monitoring. This comprehensive overview of formaldehyde concentrations is crucial for assessing occupational exposures in population-based epidemiological studies. Chronic diseases, such as cancer or neurodegenerative diseases, have long latencies and require historical exposure assessment. Therefore, our detailed historical quantitative data on formaldehyde exposure will support the approximate formaldehyde exposure assessment for people who previously worked in relevant industries during those periods.

Industries involving the production of formaldehyde are present nearly all across China (Tang et al. 2009). Although over 20,447 individual assessments were extracted for this review, covering 76% of regions in China, important data from other areas may still be missing due to regional disparities in industry distribution.

Different sampling and analytical methods were employed over the decades covered. In this study, pooled exposure concentrations for each occupation and industry combined both area and personal exposure measurements, which may not fully capture workers’ actual exposure levels. Personal samplers are typically positioned closer to the worker, whereas area samplers might be placed near formaldehyde sources, leading to differences in measured concentrations. Ideally, the impact of different sampling and analytical methods would be quantified. However, this was not possible because most of the concentrations we abstracted from the Chinese literature did not specify how specifically they were sampled and analysed. Instead, most literature just referred to the applicable standards in effect in the sampling year. These methodological variations can be influenced by factors such as the specific sampler used, the placement of the sampler, the habitual movement of the worker, other environmental conditions, and analytical methods. The latter ranged from AHMT Spectrophotometry and Phenol Reagent Spectrophotometry to Gas Chromatography (GC) and Photoelectric Photometry (**Supplementary Material 3**). Another consideration is the inclusion of indoor air quality assessments in occupational settings such as hotels, hair salons, and offices. While this expanded the dataset, subtle differences in sampling methods may affect comparability. Moreover, the pooled weighted mean concentrations combined individual measurements and summary statistics such as means, medians, or ranges. One limitation of this approach is the estimation of AMs from incomplete or variable summary statistics, which may introduce bias. Additionally, although applying weights based on sample size helps to reduce the influence of studies with fewer measurements, this approach may also skew results by disproportionately reflecting large datasets from specific industries or job titles rather than capturing a broader range of occupational conditions from multiple smaller studies. Nonetheless, the overall temporal trend fitted using log-transformed task-based AMs further supported the declining trend we observed for most occupations. Advanced statistical methods, such as parameter transformation and modelling-based meta-regression, are available for pooling exposure data based on geometric means (Koh et al. 2014, 2015). However, we opted for a descriptive and transparent approach using arithmetic means due to the heterogeneity and limited detail often present in the existing data. Our straightforward method effectively addresses the current gap in occupational formaldehyde exposure assessment in China.

## Conclusion

This review of formaldehyde air concentrations in workplaces, derived from both published studies and routine occupational hazard monitoring in China, reveals a declining trend in exposure levels over the past few decades. However, certain occupations are particularly at risk of formaldehyde overexposure. These occupations include “Wood-processing-plant operators,” “Incinerator and water-treatment plant operators,” “Chemical-processing-plant operators,” “Painters,” “Pharmacologists and pathologists,” “Metal moulders and coremakers,” “Mechanical-machinery assemblers,” and “Plastic-products machine operators.” Targeted interventions and regulations are needed to mitigate formaldehyde exposure in these high-risk occupations. Furthermore, the quantitative exposure data presented in this review can serve as a valuable resource for epidemiological exposure assessments.

## Supplementary material

Supplementary material is available at *Annals of Work Exposures and Health* online.

wxaf037_suppl_Supplementary_Materials

## Data Availability

The data will be shared on reasonable request to the corresponding author.
