# Supplementary material for: Overview of historical formaldehyde occupational exposure in China
Source: Ann Work Expo Health. 2025 Jul 15;69(8):808–19. doi: 10.1093/annweh/wxaf037 (PMC12550362; doi:10.1093/annweh/wxaf037)
Supplement: wxaf037_suppl_Supplementary_Materials [file wxaf037_suppl_supplementary_materials.pdf]

Supplementary Files for "Overview of historical formaldehyde occupational exposure in China"

Jia Nie[1], Calvin B. Ge[2], Nathaniel Rothman[3], Wei Hu[3], Qing Lan[3#], Roel Vermeulen[1#], and Susan Peters[1#*]

[1] Institute for Risk Assessment Sciences, Utrecht University, Yalelaan 2, 3584 CM Utrecht, the Netherlands

[2] Netherlands Organization for Applied Scientific Research TNO, Princetonlaan 6, 3584 CB Utrecht, the Netherlands

[3] Division of Cancer Epidemiology and Genetics, National Cancer Institute, 9609 Medical Center Drive, Bethesda, Maryland 20892, United States

# These authors contributed equally

*Author to whom correspondence should be addressed. Email: s.peters@uu.nl

Supplementary Figure 1. PRISMA flowchart of the literature search and data screening process.

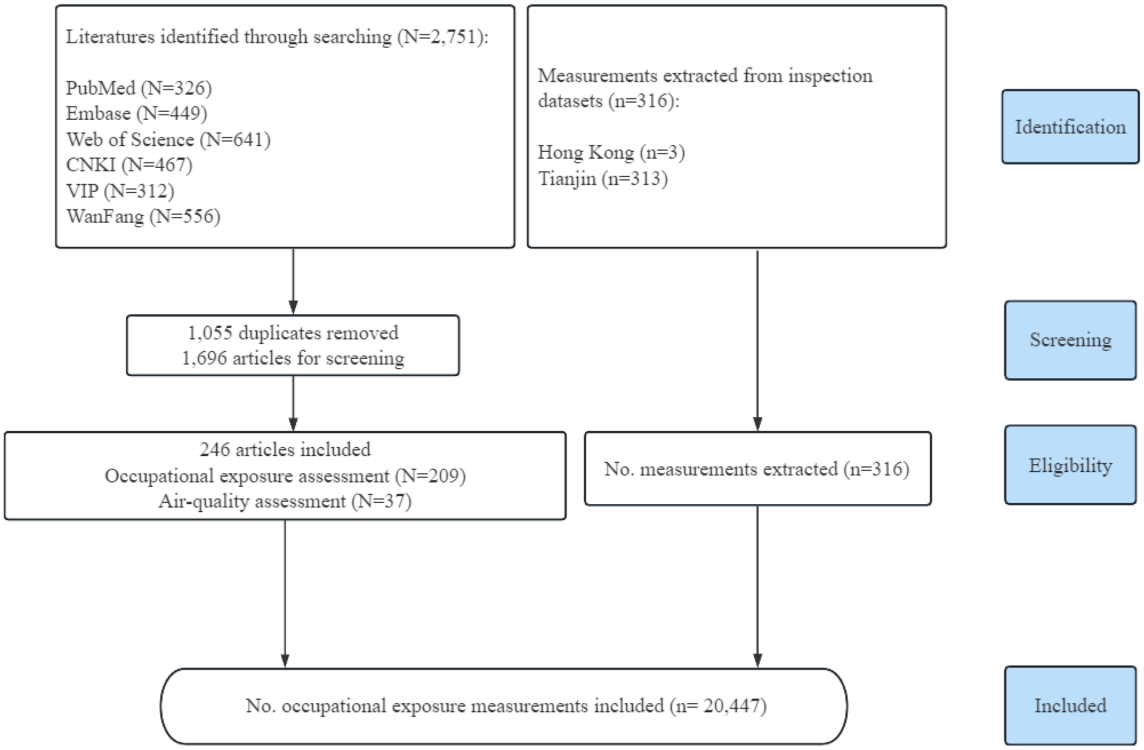

Supplementary Table 1. Information extracted from publications.

Information extracted	Description
Summary statistics	For concentrations reported as TWA, STEL, or non-specified mean concentrations in literature, we further categorized them as full-shift, task-based, or other concentrations, respectively. AM, SD, median, ranges, GM, and GSD were extracted wherever possible. Concentrations in the unit of ppm were transferred to mg/m^3 (1 ppm = 1.228 mg/m^3)
Industry	Company name or the type of industry or products they made.
Job/task	Job titles. Job tasks were extracted if job titles were not reported and then coded by occupational epidemiology researchers.
Location	The province or city.
Sample reason	Type of study for scientific publications. Industrial hygiene surveys, occupational hazard assessments, environmental air quality inspections at workplace, or other types.
Sample year	Year of sampling. When the sample year was not provided, the year of publication was used.
Type of sample	Area or personal sampling.
Number of sites measured	The number of sites selected per industry or job.
Number of samples per site	The number of samples tested per industry or job.
Number of measurements	Equals to the number of sites * the number of samples taken per site.
Sampling and analytic method	Description of sampling and analytic methods.
Working conditions	Description of the availability of ventilation systems and other related protective procedures.

Abbreviations: TWA=time weighted average, STEL=short term exposure level, AM=arithmetic mean, SD=standard deviation, GM=geometric mean, GSD=geometric standard deviation.

Supplementary Table 2. Pooled mean formaldehyde concentrations (mg/m^3) by industries in China, 1979-2023.

Industry (ISIC4)	Task-based*	No. measurements	Full-shift*	No. measurements
Extraction of crude petroleum (0610)	5.48 (0.70-5.80)	32		
Manufacture of other food products n.e.c. (1079)	0.10 (0.06-0.18)	152		
Manufacture of wines (1102)	0.06 (0.06-0.06)	30		
Spinning, weaving and finishing of textiles (1310)	0.30 (0.03-3.03)	42		
Manufacture of wearing apparel, except fur apparel (1410)	0.37 (0.08-6.14)	174		
Tanning and dressing of leather; dressing and dyeing of fur (1511)	0.09 (0.08-0.10)	90		
Manufacture of veneer sheets and wood-based panels (1621)	0.69 (0.02-4.98)	3645	0.62 (0.09-1.48)	20
Printing (1811)	0.07 (0.03-0.18)	58		
Manufacture of basic chemicals (2011)	0.68 (0.00-7.80)	300	5.15 (0.15-8.80)	21
Manufacture of plastics and synthetic rubber in primary forms (2013)	1.10 (0.03-9.90)	226	5.19 (1.05-8.70)	6
Manufacture of pesticides and other agrochemical products (2021)	1.29 (0.02-2.70)	26		
Manufacture of paints, varnishes and similar coatings, printing ink and mastics (2022)	0.12 (0.01-0.88)	109	0.15 (0.07-0.52)	22
Manufacture of soap and detergents, cleaning and polishing preparations, perfumes and toilet preparations (2023)	0.19 (0.01-1.69)	254	1.55 (0.98-1.57)	405
Manufacture of other chemical products n.e.c. (2029)	0.27 (0.07-1.14)	100		
Manufacture of glass and glass products (2310)	1.37 (0.04-2.31)	32		
Manufacture of basic iron and steel (2410)	1.48 (0.11-1.89)	331	0.54 (0.48-0.60)	6
Treatment and coating of metals; machining (2592)	0.02 (0.02-0.02)	72		
Manufacture of other fabricated metal products n.e.c. (2599)	0.45 (0.33-0.63)	38		
Manufacture of electronic components and boards (2610)	0.09 (0.00-0.73)	238		
Manufacture of office machinery and equipment (except computers and peripheral equipment) (2817)	1.30 (1.30-1.30)	38		
Manufacture of motor vehicles (2910)	0.28 (0.02-1.99)	47	0.28 (0.03-4.70)	533
Manufacture of parts and accessories for motor vehicles (2930)	0.69 (0.02-8.30)	82	0.26 (0.07-0.64)	68
Manufacture of furniture (3100)	0.29 (0.00-3.33)	724	0.22 (0.07-0.32)	17
Manufacture of sports goods (3230)	0.32 (0.32-0.32)	34		
Sewerage (3700)	0.97 (0.04-1.56)	834		
Plumbing, heat and air-conditioning installation (4322)	0.03 (0.03-0.03)	896		
Building completion and finishing (4330)	0.59 (0.29-1.27)	1696		
Wholesale of textiles, clothing and footwear (4641)	0.17 (0.14-0.18)	381		
Wholesale of electronic and telecommunications equipment and parts (4652)	0.11 (0.10-0.11)	102		

Industry (ISIC4)	Task-based*	No. measurements	Full-shift*	No. measurements
Other retail sale in non-specialized stores (4719)	0.10 (0.01-1.11)	366		
Retail sale of electrical household appliances, furniture, lighting equipment and other household articles in specialized stores (4759)	0.16 (0.05-0.28)	452		
Retail sale of second-hand goods (4774)	0.05 (0.04-0.15)	358		
Passenger rail transport, interurban (4911)	0.01 (0.00-0.03)	640		
Urban and suburban passenger land transport (4921)	0.09 (0.02-0.52)	33		
Freight air transport (5120)	0.01 (0.01-0.01)	165		
Short term accommodation activities (5510)	0.06 (0.03-0.09)	1537		
Restaurants and mobile food service activities (5610)	0.06 (0.01-0.09)	87		
Combined office administrative service activities (8211)	0.06 (0.02-0.27)	1098	0.07 (0.07-0.07)	40
Other human health activities (8690)	0.86 (0.02-12.0)	561	0.51 (0.51-0.51)	2
Creative, arts and entertainment activities (9000)	0.06 (0.06-0.06)	267		
Library and archives activities (9101)	0.07 (0.00-0.08)	230		
Activities of cultural or recreational associations (other than sports or games) (9497)	0.07 (0.07-0.07)	38		
Hairdressing and other beauty treatment (9602)	0.05 (0.02-0.10)	1383		

Notes: only industries with at least 25 measurements were listed in the table.
* Weighted mean concentrations (minimum-maximum) were calculated for all measurements from both publications and representative measurements, and the minimum and maximum of the reported concentrations were shown in brackets.

Supplementary Table 3. Pooled mean formaldehyde concentrations (mg/m^3) by occupations and industries in China, 1979-2023.

Job Title (ISCO88)/Industry (ISIC4)	Task-based*	No. measurements	Full-shift*	No. measurements
Chemists (2113)				
Manufacture of pharmaceuticals, medicinal chemical and botanical products (2100)	0.16 (0.16-0.16)	3		
Life science and health professionals (2200)				
Other human health activities (8690)	0.26 (0.14-1.79)	49		
Pharmacologists, pathologists and related professionals (2212)				
Other human health activities (8690)	0.92 (0.02-12.0)	512	0.51 (0.51-0.51)	2
College, university and higher education teaching professionals (2310)				
Educational support activities (8550)	3.91 (0.39-12.8)	12		
Librarians and related information professionals (2432)				
Library and archives activities (9101)	0.07 (0.00-0.08)	230		
Sculptors, Painters and Related Artists (2452)				
Service activities related to printing (1812)	0.22 (0.07-0.36)	4		
Religious professionals (2460)				
Activities of religious organizations (9491)	0.07 (0.07-0.07)	16		
Chemical engineering technicians (3116)				
Manufacture of basic chemicals (2011)	2.64 (0.88-4.39)	4		
Manufacture of paints, varnishes and similar coatings, printing ink and mastics (2022)	0.07 (0.07-0.07)	6		
Manufacture of veneer sheets and wood-based panels (1621)	0.66 (0.03-0.78)	13		
Spinning, weaving and finishing of textiles (1310)	0.19 (0.19-0.19)	1		
Life Science Technicians (3211)				
Marine fishing (0311)	0.00 (0.00-0.00)	1		
Police Inspectors and Detectives (3450)				
Public order and safety activities (8423)			0.14 (0.12-0.17)	2
Office clerks (4100)				
Combined office administrative service activities (8211)	0.06 (0.02-0.27)	1098	0.07 (0.07-0.07)	40
Manufacture of power-driven hand tools (2818)			0.31 (0.23-0.40)	2
Stock clerks (4131)				
Manufacture of furniture (3100)	0.22 (0.22-0.22)	3		
Manufacture of pharmaceuticals, medicinal chemical and botanical products (2100)	0.05 (0.05-0.05)	1		
Manufacture of veneer sheets and wood-based panels (1621)	0.62 (0.22-1.23)	93		
Manufacture of wearing apparel, except fur apparel (1410)	0.49 (0.49-0.49)	1		
Spinning, weaving and finishing of textiles (1310)	1.62 (1.62-1.62)	2		
Personal and protective services workers (5100)				
Activities of cultural or recreational associations (other than sports or games) (9497)	0.07 (0.07-0.07)	38		
Other amusement and recreation activities n.e.c. (9329)	0.13 (0.13-0.13)	12		
Travel attendants and travel stewards (5111)				
Freight air transport (5120)	0.01 (0.01-0.01)	165		

Job Title (ISCO88)/Industry (ISIC4)	Task-based*	No. measurements	Full-shift*	No. measurements
Unknown (NA)	0.07 (0.07-0.07)	28		
Passenger rail transport, interurban (4911)	0.01 (0.00-0.03)	640		
Waiters, waitresses and bartenders (5123)				
Restaurants and mobile food service activities (5610)	0.06 (0.01-0.09)	87		
Hairdressers, barbers, beauticians and related workers (5141)				
Hairdressing and other beauty treatment (9602)	0.05 (0.02-0.10)	1383		
Stall and market salespersons (5230)				
Other retail sale in non-specialized stores (4719)	0.10 (0.01-1.11)	366		
Retail sale of electrical household appliances, furniture, lighting equipment and other household articles in specialized stores (4759)	0.16 (0.05-0.28)	452		
Retail sale of second-hand goods (4774)	0.05 (0.04-0.15)	358		
Wholesale of electronic and telecommunications equipment and parts (4652)	0.11 (0.10-0.11)	102		
Wholesale of textiles, clothing and footwear (4641)	0.17 (0.14-0.18)	381		
Stone splitters, cutters and carvers (7113)				
Cutting, shaping and finishing of stone (2396)	0.72 (0.72-0.72)	24		
Manufacture of articles of concrete, cement and plaster (2395)	1.10 (1.10-1.10)	1		
Carpenters and joiners (7124)				
Building of ships and floating structures (3011)	0.04 (0.04-0.04)	6		
Manufacture of furniture (3100)	0.36 (0.36-0.36)	1	0.30 (0.30-0.30)	9
Building finishers and related trade workers not elsewhere classified (713)				
Building completion and finishing (4330)	0.30 (0.29-1.27)	1094		
Plumbers and pipe fitters (7136)				
Plumbing, heat and air-conditioning installation (4322)	0.03 (0.03-0.03)	896		
Painters and related workers (7141)				
Building completion and finishing (4330)	1.12 (1.12-1.12)	602		
Varnishers and related painters (7142)				
Maintenance and repair of motor vehicles (4520)	0.03 (0.03-0.04)	2		
Manufacture of bodies (coachwork) for motor vehicles; manufacture of trailers and semi-trailers (2920)			0.13 (0.07-0.41)	18
Manufacture of chemicals and chemical products (2000)			0.16 (0.07-0.30)	18
Manufacture of furniture (3100)	0.10 (0.00-0.16)	18		
Manufacture of motor vehicles (2910)	0.39 (0.05-1.70)	9	0.38 (0.07-4.70)	131
Manufacture of parts and accessories for motor vehicles (2930)			0.28 (0.13-0.50)	7
Manufacture of veneer sheets and wood-based panels (1621)	0.40 (0.40-0.40)	1		
Unknown (NA)	0.69 (0.69-0.69)	10		
Metal moulders and coremakers (7211)				
Maintenance and repair of motor vehicles (4520)	0.04 (0.04-0.04)	1		
Manufacture of basic iron and steel (2410)	1.49 (1.00-1.89)	329		
Manufacture of motor vehicles (2910)	0.85 (0.16-1.99)	8		

Job Title (ISCO88)/Industry (ISIC4)	Task-based*	No. measurements	Full-shift*	No. measurements
Manufacture of parts and accessories for motor vehicles (2930)	0.56 (0.16-0.59)	29	0.30 (0.07-0.56)	23
Manufacture of structural metal products (2511)	0.25 (0.25-0.25)	1		
Blacksmiths, tool-makers and related trades workers (7220)				
Manufacture of metal-forming machinery and machine tools (2822)	0.25 (0.25-0.25)	1		
Tool-makers and related workers (7222)				
Manufacture of motor vehicles (2910)			0.23 (0.23-0.23)	1
Metal wheel-grinders, polishers and tool sharpeners (7224)				
Manufacture of motor vehicles (2910)	0.25 (0.25-0.25)	3	0.27 (0.07-0.39)	6
Manufacture of parts and accessories for motor vehicles (2930)	0.19 (0.19-0.19)	1		
Silk-screen, block and textile printers (7346)				
Manufacture of builders' carpentry and joinery (1622)	0.08 (0.08-0.08)	6		
Wood treaters season and preserve wood (7421)				
Manufacture of furniture (3100)	0.34 (0.34-0.34)	16		
Woodworking-machine setters and setter-operators (7423)				
Manufacture of furniture (3100)	0.24 (0.08-0.26)	13		
Upholsterers and related workers (7437)				
Manufacture of parts and accessories for motor vehicles (2930)	0.17 (0.02-0.32)	36		
Shoe-makers and related workers (7442)				
Manufacture of footwear (1520)	0.41 (0.32-0.63)	5		
Well drillers and borers and related workers (8113)				
Extraction of crude petroleum (0610)	5.48 (0.70-5.80)	32		
Support activities for petroleum and natural gas extraction (0910)	0.06 (0.00-0.08)	15		
Metal-processing plant operators (812)				
Manufacture of basic iron and steel (2410)	0.11 (0.11-0.11)	2		
Manufacture of structural metal products (2511)	0.01 (0.01-0.01)	2		
Metal melters, casters and rolling-mill operators (8122)				
Manufacture of motor vehicles (2910)			0.60 (0.56-0.67)	3
Metal heat-treating-plant operators (8123)				
Manufacture of basic iron and steel (2410)			0.54 (0.48-0.60)	6
Manufacture of motor vehicles (2910)			0.11 (0.07-0.20)	3
Manufacture of structural metal products (2511)	0.01 (0.01-0.02)	12		
Metal drawers and extruders operate (8124)				
Manufacture of motor vehicles (2910)			0.11 (0.07-0.14)	3
Glass and ceramics kiln and related machine operators (8131)				
Manufacture of glass and glass products (2310)	1.37 (0.04-2.31)	32		
Wood-processing-plant operators (8141)				
Manufacture of furniture (3100)	0.26 (0.04-0.90)	90		
Manufacture of veneer sheets and wood-based panels (1621)	0.69 (0.02-4.98)	3479	0.62 (0.09-1.48)	20
Chemical-processing-plant operators (8150)				

Job Title (ISCO88)/Industry (ISIC4)	Task-based*	No. measurements	Full-shift*	No. measurements
Manufacture of basic chemicals (2011)	0.67 (0.01-2.51)	229		
Manufacture of fertilizers and nitrogen compounds (2012)	0.14 (0.03-0.20)	6		
Manufacture of other chemical products n.e.c. (2029)	0.19 (0.07-0.31)	72		
Manufacture of paints, varnishes and similar coatings, printing ink and mastics (2022)	0.10 (0.01-0.28)	71		
Manufacture of plastics and synthetic rubber in primary forms (2013)	5.00 (5.00-5.00)	2		
Manufacture of soap and detergents, cleaning and polishing preparations, perfumes and toilet preparations (2023)	0.63 (0.10-1.69)	54	1.55 (0.98-1.57)	405
Crushing-, grinding- and chemical-mixing-machinery (8151)				
Manufacture of basic chemicals (2011)	0.21 (0.03-0.52)	17	1.87 (1.87-1.87)	2
Manufacture of chemicals and chemical products (2000)	0.04 (0.04-0.04)	1		
Manufacture of other chemical products n.e.c. (2029)	0.20 (0.20-0.20)	1		
Manufacture of paints, varnishes and similar coatings, printing ink and mastics (2022)	0.17 (0.07-0.33)	24	0.18 (0.07-0.52)	15
Manufacture of parts and accessories for motor vehicles (2930)			0.35 (0.07-0.64)	2
Chemical-heat-treating-plant operators (8152)				
Manufacture of basic chemicals (2011)			3.12 (3.12-3.12)	8
Manufacture of chemicals and chemical products (2000)	0.02 (0.02-0.02)	1		
Chemical-filtering- and separating-equipment operators (8153)				
Manufacture of basic chemicals (2011)	0.47 (0.47-0.47)	2		
Still and reactor operators (except petroleum and natural gas) (8154)				
Manufacture of basic chemicals (2011)	1.06 (0.00-7.80)	25	7.84 (0.15-8.80)	10
Manufacture of paints, varnishes and similar coatings, printing ink and mastics (2022)	0.26 (0.26-0.26)	3		
Manufacture of plastics and synthetic rubber in primary forms (2013)	5.98 (2.03-9.10)	8		
Incinerator, water-treatment and related plant operators (8163)				
Electric power generation, transmission and distribution (3510)	0.25 (0.25-0.25)	1		
Manufacture of parts and accessories for motor vehicles (2930)	0.20 (0.20-0.20)	1		
Sewerage (3700)	0.97 (0.04-1.56)	834		
Spinning, weaving and finishing of textiles (1310)	0.11 (0.11-0.11)	1		
Treatment and disposal of hazardous waste (3822)	0.00 (0.00-0.00)	15		
Cement and other mineral products machine operators (8212)				
Manufacture of other non-metallic mineral products n.e.c. （2399）	0.25 (0.25-0.25)	1		
Pharmaceutical-and toiletry-products machine operators (8221)				
Manufacture of pesticides and other agrochemical products (2021)	1.29 (0.02-2.70)	26		
Manufacture of pharmaceuticals, medicinal chemical and botanical products (2100)	0.26 (0.04-0.53)	7		
Manufacture of soap and detergents, cleaning and polishing preparations, perfumes and toilet preparations (2023)	0.07 (0.01-0.25)	200		
Metal finishing-, plating- and coating-machine operators (8223)				
Treatment and coating of metals; machining (2592)	0.02 (0.02-0.02)	72		
Rubber-products machine operators (8231)				
Manufacture of parts and accessories for motor vehicles (2930)	0.05 (0.02-0.09)	10		

Job Title (ISCO88)/Industry (ISIC4)	Task-based*	No. measurements	Full-shift*	No. measurements
Manufacture of plastics and synthetic rubber in primary forms (2013)	0.57 (0.13-2.00)	76		
Manufacture of sports goods (3230)	0.32 (0.32-0.32)	34		
Plastic-products machine operators (8232)				
Manufacture of motor vehicles (2910)	0.02 (0.02-0.02)	18		
Manufacture of other chemical products n.e.c. (2029)	0.48 (0.23-1.14)	27		
Manufacture of other electrical equipment (2790)	0.07 (0.07-0.07)	1		
Manufacture of parts and accessories for motor vehicles (2930)	0.10 (0.10-0.10)	1	0.23 (0.13-0.37)	36
Manufacture of plastics and synthetic rubber in primary forms (2013)	1.00 (0.03-9.90)	138	5.19 (1.05-8.70)	6
Manufacture of wearing apparel, except fur apparel (1410)	0.45 (0.10-1.68)	72		
Wood-products machine operators (8240)				
Manufacture of furniture (3100)	0.26 (0.05-1.26)	542	0.07 (0.07-0.07)	3
Manufacture of veneer sheets and wood-based panels (1621)	2.31 (1.17-3.80)	18		
Printing-machine operators (8251)				
Printing (1811)	0.07 (0.03-0.18)	58		
Service activities related to printing (1812)	0.56 (0.56-0.56)	11		
Textile-, fur- and leather-products machine operators (8260)				
Manufacture of games and toys (3240)	0.25 (0.25-0.25)	1		
Fibre-preparing, spinning- and winding-machine operators (8261)				
Manufacture of other fabricated metal products n.e.c. (2599)	0.45 (0.33-0.63)	38		
Manufacture of other textiles n.e.c. (1399)	0.50 (0.50-0.50)	2		
Manufacture of wearing apparel, except fur apparel (1410)	3.60 (3.60-3.60)	1		
Preparation and spinning of textile fibres (1311)	1.56 (0.22-5.59)	24		
Spinning, weaving and finishing of textiles (1310)	1.62 (0.58-3.03)	4		
Sewing-machine operators (8263)				
Manufacture of wearing apparel, except fur apparel (1410)	3.11 (0.08-6.14)	2		
Bleaching-, dyeing- and cleaning-machine operators (8264)				
Spinning, weaving and finishing of textiles (1310)	0.08 (0.03-0.09)	34		
Tanning and dressing of leather; dressing and dyeing of fur (1511)	0.09 (0.08-0.10)	90		
Food and related products machine operators (8270)				
Manufacture of other food products n.e.c. (1079)	0.09 (0.06-0.12)	144		
Brewers, wine and other beverage machine operators (8278)				
Manufacture of wines (1102)	0.06 (0.06-0.06)	30		
Mechanical-machinery assemblers (8281)				
Manufacture of motor vehicles (2910)	0.17 (0.17-0.17)	9	0.24 (0.03-0.56)	386
Manufacture of parts and accessories for motor vehicles (2930)	8.30 (8.30-8.30)	4		
Electronic-equipment assemblers (8283)				
Manufacture of consumer electronics (2640)	0.49 (0.49-0.49)	1		
Manufacture of electric lighting equipment (2740)	0.24 (0.24-0.24)	12		
Manufacture of electronic components and boards (2610)	0.09 (0.00-0.73)	238		

Job Title (ISCO88)/Industry (ISIC4)	Task-based*	No. measurements	Full-shift*	No. measurements
Manufacture of office machinery and equipment (except computers and peripheral equipment) (2817)	1.30 (1.30-1.30)	38		
Metal-, rubber- and plastic-products assemblers (8284)				
Manufacture of wearing apparel, except fur apparel (1410)	0.18 (0.18-0.18)	96		
Wood and related products assemblers (8285)				
Manufacture of furniture (3100)	0.74 (0.03-3.33)	41	0.18 (0.12-0.32)	5
Other machine operators and assemblers (8290)				
Manufacture of basic chemicals (2011)	0.46 (0.01-2.00)	23	1.20 (1.20-1.20)	1
Manufacture of paints, varnishes and similar coatings, printing ink and mastics (2022)	0.27 (0.11-0.88)	5	0.09 (0.07-0.19)	7
Manufacture of plastics and synthetic rubber in primary forms (2013)	4.50 (4.50-4.50)	2		
Car, taxi and van drivers (8322)				
Urban and suburban passenger land transport (4921)	0.07 (0.03-0.08)	21		
Bus and tram drivers (8323)				
Urban and suburban passenger land transport (4921)	0.12 (0.02-0.52)	12		
Lifting-truck operators (8334)				
Manufacture of fertilizers and nitrogen compounds (2012)	0.03 (0.03-0.03)	1		
Helpers and cleaners in offices, hotels and other establishments (9132)				
Short term accommodation activities (5510)	0.06 (0.03-0.09)	1537		
Hand-launderers and pressers (9133)				
Manufacture of wearing apparel, except fur apparel (1410)	1.85 (0.91-2.80)	2		
Construction and maintenance labourers (9312)				
Construction of roads and railways (4210)	0.16 (0.16-0.16)	2		
Hand packers and other manufacturing labourers (9322)				
Manufacture of other food products n.e.c. (1079)	0.18 (0.18-0.18)	8		
Manufacture of veneer sheets and wood-based panels (1621)	0.48 (0.07-0.75)	41		
Unknown (NA)				
Building of ships and floating structures (3011)	0.62 (0.62-0.62)	11		
Creative, arts and entertainment activities (9000)	0.06 (0.06-0.06)	267		
Unknown (NA)	0.07 (0.01-0.50)	510	0.03 (0.01-0.11)	498

* Weighted mean concentrations (minimum-maximum) were calculated for all measurements from both publications and representative measurements, and the minimum and maximum of the reported concentrations were shown in brackets.

Supplementary Table 4. Pooled mean formaldehyde concentration (mg/m^3) by different regions in China, 1979-2023.

Location	Task-based*	No. measurements	Full-shift*	No. measurements
Anhui	0.23 (0.04-3.07)	188		
Beijing	0.16 (0.02-1.26)	1099		
Chongqing	0.28 (0.00-3.03)	160	0.24 (0.03-0.56)	366
Fujian	0.50 (0.00-1.26)	181		
Gansu	3.35 (0.00-12.8)	14		
Guangdong	0.76 (0.00-4.98)	1651	0.83 (0.03-8.80)	886
Guangxi	0.46 (0.05-2.39)	100		
Guizhou	0.34 (0.34-0.34)	2		
Hebei	0.55 (0.12-3.03)	273		
Heilongjiang	0.36 (0.02-0.72)	52		
Henan	0.43 (0.01-5.80)	1335		
Hong Kong	0.06 (0.00-6.14)	979	0.07 (0.07-0.07)	40
Hubei	0.68 (0.01-3.80)	1274	0.35 (0.01-0.98)	59
Hunan	1.44 (0.02-9.10)	121		
Jiangsu	0.14 (0.02-5.59)	3459		
Jiangxi	0.85 (0.02-2.25)	308		
Jilin	0.21 (0.03-1.88)	90		
Liaoning	0.62 (0.03-1.30)	93		
Shandong	0.26 (0.00-3.60)	3030		
Shanghai	0.24 (0.00-2.51)	969	4.48 (0.15-8.70)	9
Shanxi	1.24 (0.01-8.30)	30		
Sichuan	1.87 (0.02-6.20)	108		
Tianjin	0.91 (0.09-9.90)	55	0.29 (0.07-4.70)	300
Xinjiang	0.72 (0.03-12.0)	145		
Yunnan	0.08 (0.08-0.16)	52		
Zhejiang	0.75 (0.02-5.47)	1691	0.35 (0.15-0.60)	18
Several region or unknown	0.18 (0.01-5.80)	1310		

* Weighted mean concentrations (minimum-maximum) were calculated for all measurements from both publications and representative measurements, and the minimum and maximum of the reported concentrations were shown in brackets.

Supplementary Table 5. Pooled mean formaldehyde concentrations (mg/m^3) by sampling type in China, 1979-2023.

Data source	Year period	Task-based*	No. measurements	Full-shift*	No. measurements
area	1990 and earlier	1.60 (0.15-6.14)	500		
	1991-2000	1.12 (0.00-12.8)	267	0.24 (0.01-0.98)	99
	2001-2010	0.24 (0.00-9.10)	6682	0.45 (0.07-4.70)	110
	2011 and after	0.41 (0.00-12.0)	11305	0.23 (0.03-0.67)	576
personal	1990 and earlier			4.48 (0.15-8.70)	9
	2001-2010	1.12 (0.25-1.99)	6	0.83 (0.03-8.80)	884
	2011 and after	0.08 (0.08-0.08)	9		

* Weighted mean concentrations (minimum-maximum) were calculated for all measurements from both publications and representative measurements, and the minimum and maximum of the reported concentrations were shown in brackets.

Supplementary Material 1: Search terms used in different Chinese database.

Number of literatures found in CNKI: 467

篇关摘=(((职业 + 工业 + 工作) * 暴露) + (工作环境 * 空气浓度) + (职业卫生) + (职业健康) + (职业风险) + (职业危害)) * (甲醛 + 福尔马林 + 福美林 + 蚁醛) [模糊]

Number of literatures found in VIP：312

篇关摘=(((职业 + 工业 + 工作) * 暴露) + (工作环境 * 空气浓度) + (职业卫生) + (职业健康) + (职业风险) + (职业危害)) * (甲醛 + 福尔马林 + 福美林 + 蚁醛) [模糊]

Number of literatures found in Wanfang: 556

主题:((职业 AND 暴露) OR (工业 AND 暴露) OR (工作 AND 暴露) OR (工作环境 AND 空气浓度) OR (职业卫生) OR (职业健康) OR (职业风险) OR (职业危害)) and 全部:(甲醛 OR 福尔马林 OR 福美林 OR 蚁醛) [模糊]

Supplementary Material 2: Publications used for concentration extraction.

1. 安玉; 何作力; 宋月. 2003-2013 年大连某办公设备制造企业职业病危害因素调查分析[J]. 中国卫生工程学. 2015.

2. 戎艳; 吴越芾; 张静波; 郭孔荣; 孙道远. 2014 年上海浦东新区某黑色金属铸造企业职业病危害关键控制点分析[J]. 职业与健康. 2016.

3. 陈凤琼; 李小平; 冉瑞红. 2015-2020 年重庆市汽车制造业重点职业病危害因素监测结果分析[J]. Occupation and Health. 2022.

4. 徐璐; 朱贺坤; 罗艺丰; 王志行. 2016 年佛山市某涂料化工企业职业病危害现状调查[J]. 绿色科技. 2017.

5. 胡志平; 丁士昆; 吴成峰; 张乾驰. 2017 年铜陵市某铁路机车配件制造企业职业病危害现状[J]. 职业与健康. 2018.

6. 翟和亮; 陈栋; 罗慧敏; 李玉. 2018-2019 年六安市宾馆室内空气卫生状况及影响因素分析[J]. Journal of Preventive Medicine Information. 2021.

7. 唐慧晶; 刘保峰; 秦汝男; 李旭东; 刘涛; 曾强. 2018-2020 年天津市某综合性医院病理科化学性职业病危害因素调查[J]. 职业与健康. 2022.

8. 曲鹏飞; 李世慧; 冯维春. HACCP 体系在某聚甲醛生产企业职业病危害控制效果评价中的应用[J]. 职业与健康. 2017.

9. 帅志勇; 葛怡琛; 阮小林; 谢玉旋; 赖关朝; 刘松旺; 唐小江. 三聚氰胺树脂生产工厂甲醛接触水平调查[J]. CHINA OCCUPATIONAL MEDICINE. 2010.

10. 王凯; 陈健; 陈良. 上海市 3 家氨纶纤维生产企业职业卫生现状调查[J]. 上海预防医学. 2015.

11. 高翔; 白志鹏; 游燕; 苗娟; 刘冰. 不同室内环境空气中挥发性有机物的暴露水平及其对健康的影响[J]. 环境与健康杂志. 2006.

12. 顾永权; 王爱红; 毛荷明; 胡向前; 冷朋波; 苗超. 两家黑色金属铸造企业职业健康风险评估结果分析[J]. 预防医学. 2021.

13. 曹素红. 两种职业健康风险评估模型在上海市奉贤区某汽车零部件制造企业中的运用[J]. 职业与健康. 2018.

14. 张美辨, 王爱红； 冷朋波； 边国林； 李晓海； 毛国传. 两种风险评估模型在木质家具制造企业职业健康风险评估中的应用[J]. 中华劳动卫生职业病杂志. 2016.

15. 顾海; 刘盛. 乌鲁木齐市医疗机构病理科甲醛、二甲苯水平调查[J]. 中国职业医学. 2018.

16. 刘琳琳; 葛蕴珊; 李兰; 张传桢. 乘用车内空气质量健康风险评估[J]. Research of Environmental Sciences. 2016.

17. 邵华, 程学美； 赵敬； 冯斌； 温培娥. 乙醛脱氢酶 2 和细胞色素 P4502E1 基因多态性与甲醛职业危害易感性的关系[J]. 中华预防医学杂志. 2008.

18. 陈琛, 马玉英； 李洪； 许东. 人造板工人外周血淋巴细胞微核率的观察[J]. 河南预防医学杂志. 1995.

19. 贾力; 刘晓燕; 徐国锋. 人造板材制造人员血清中 SOD 活力及 MDA 含量分析[J]. 河南预防医学杂志. 2015.
20. 何中凯; 张寅平. 人造板车间内工人甲醛暴露的健康风险评估[J]. 建筑科学. 2010.
21. 陶羡梅；刘纯华；赵小琴. 低浓度甲醛对工人职业危害调查[J]. 工业卫生与职业病. 1990. 1000-7164
22. 叶恩林; 邓雪凝; 劳少泉; 徐娜; 黄应和; 陈健安. 佛山市某复合肥料厂职业病危害控制效果评价[J]. 职业与健康. 2013.
23. 缪明霞; 张雪梅; 张晓丽. 佳木斯市某汽车服务有限公司维修车间职业病危害现状调查[J]. 河南预防医学杂志. 2019.
24. 翟和亮; 陈栋; 罗慧敏; 李玉. 六安市公共场所室内空气中甲醛污染对从业人员的健康风险评价[J]. 职业与健康. 2020.
25. 王力民; 木拉提; 左虹; 李雪; 李向东. 兰新第二双线高速铁路动车组车内环境综合评价[J]. 铁路节能环保与安全卫生. 2015.
26. 张静; 张秋玲; 李焕焕; 周桂侠. 凹印制版过程中的尘毒危害识别与关键控制点分析[J]. 中国工业医学杂志. 2014.
27. 马立新. 分析公共场所游离甲醛来源及对人体危害和有效控制[J]. World Health Digest. 2014.
28. 马小惠; 张杰; 黄志超; 于亚鹭; 赵新伟; 努尔比耶·约麦尔; 吴军. 办公场所空气污染水平及与人群早期刺激症状关系的调查[J]. Journal of Xinjiang Medical University. 2017.
29. 王鹏; 张玉. 北京市 34 家医院职业病危害因素接触水平现状调查[J]. 职业与健康. 2022.
30. 孙冉; 赵永梅; 马璨; 左昕; 王磊; 沈成钢. 北京市 50 家医疗机构重点职业危害及职业健康管理现状调查[J]. 中国工业医学杂志. 2021.
31. 张金萍; 陈文军; 杜鹏宇. 北京市公共场所室内甲醛浓度水平分析[J]. 环境污染与防治. 2015.
32. 李晓光; 邓喆轩; 王雪涛. 北京市某家具制造厂职业病危害因素调查[J]. 职业与健康. 2021.
33. 刘丽霞; 谭佳红; 曹建彪. 北京市某密度板制造企业 MDF 车间职业病危害调查[J]. 职业与健康. 2017.
34. 马奎; 佟林全; 王雪涛. 北京市某木质家具厂职业病危害现状与对策[J]. 职业与健康. 2019.
35. 伍家琪; 王海椒; 贾晋阳. 北京市某涂料生产企业职业病危害现状调查[J]. 职业与健康. 2020.
36. 李静芸; 杨汉彬; 马奎; 贾晋阳; 徐洋; 佟林全; 王雪涛. 北京市某生物医药企业研发部门实验室化学有害因素职业病危害现状[J]. 职业与健康. 2020.
37. 邹磊. 北京市某织染企业职业病危害现状评价[J]. 职业与健康. 2016.
38. 周国伟; 胡在方; 张志旭; 周亮; 张晓辉. 北京市顺义区 20 家家具企业职业卫生状况调查[J]. 中国卫生检验杂志. 2011.
39. 顾海; 刘盛. 医学解剖实验室师生甲醛职业接触水平与通风效果调查分析[J]. 公共卫生与预防医学. 2018.

40. 彭巨成; 谌阿璟; 林祥吉. 半定量综合指数法在深圳市某家具企业职业健康风险评估中的应用[J]. 职业与健康. 2019.
41. 黄吉; 段平宁; 黄翔; 聂传丽; 麦骊风. 南宁市某胶合板企业职业健康风险评估[J]. 职业与健康. 2022.
42. 黄文琪; 徐宇萍; 谢金明; 刘小安; 刘永泉. 危害分析与关键点控制原理在木质家具制造企业职业病危害防控中的应用[J]. 工业卫生与职业病. 2021.
43. 左弘; 何家禧; 李天正; 邓敏; 丘海丽; 黄红英. 危险废物综合处理行业职业病危害防护效果的调查[J]. 职业与健康. 2012.
44. 王政; 张金萍; 张佳琳; 王霁月; 李冠群; 杨怀宇. 商业类不同功能公共场所室内甲醛浓度水平及健康风险评价[J]. 建筑科学. 2021.
45. 王娅囡, 李林； 郑昀； 王芳； 牛东升. 垃圾焚烧发电企业职业危害现况调查[J]. 中华劳动卫生职业病杂志. 2020.
46. 张建华, 崔力争. 大医院病理科甲醛污染危害的评价[J]. 中国卫生工程学. 1995.
47. 王艳玲. 天津市某汽车冲压模具铸件公司职业病危害因素检测与评价[J]. 职业与健康. 2016.
48. 元培红; 陈文斌; 赵艳君. 太原市某家具厂职业病危害因素调查[J]. 职业与健康. 2013.
49. 韩世丽; 邱劲松. 定量和半定量评估法评估某家具制造企业职业健康风险[J]. 职业卫生与病伤. 2020.
50. 钟永根, 封蔚莹； 陈志敏； 葛国兴. 室内装修油漆作业环境污染及其对作业人员健康的影响[J]. 中华劳动卫生职业病杂志. 2013.
51. 杨章萍; 曹坚忠; 张旭慧; 姜彩霞; 印晓虹; 盛萍. 家具制造业职业病危害因素检测与分析[J]. 中国卫生检验杂志. 2008.
52. 栾俞清; 张美辨; 邹华; 全长健. 家具制造企业半定量风险评估方法优化及应用研究[J]. 预防医学. 2017.
53. 何作顺; 宋正蕊; 张态; 李蕴成; 章丽娟; 陈红云. 家具城内空气中甲醛水平对人体健康影响分析[J]. 现代预防医学. 2006.
54. 魏海燕. 家具销售市场室内甲醛污染现状及其防治[J]. Co-operative Economy& Science. 2012.
55. 耿月华; 孟宪辉; 李宪成; 路广英. 密度板行业职业危害及对健康的影响[J]. 职业与健康. 2004. 1004-1257
56. 张美辨, 刘弢； 张鹏； 李辉； 张传会； 马力. 小型家具制造企业风险评估中化学物质半定量风险评估法的应用[J]. 中华劳动卫生职业病杂志. 2018.
57. 靳建超; 李俊春; 刘梅; 赵冰; 陈恩泽. 山东省农药企业主要职业病危害因素调查分析[J]. 中国卫生工程学. 2016.
58. 张茜; 李广益; 侯学文; 汪洋; 于平. 山东省某汽车制造有限公司年产 10 万辆新能源乘用车项目职业病危害控制效果评价[J]. 职业与健康. 2019.

59. 尼佳乐; 赵相云. 平顶山市 7 家医院病理科室内甲醛、二甲苯浓度调查[J]. 中国卫生工程学. 2020.
60. 宋敏丽. 广东省佛山市 3 家水性涂料生产企业职业病危害调查及作业分级[J]. 广东化工. 2018.
61. 王伟鸿. 广州市公共场所室内空气中甲醛浓度水平的分析[J]. SOUTH CHINA JOURNAL OF PREVENTIVE MEDICINE. 2004.
62. 马炜钰; 谭夏优. 广州市木质家具制造行业主要职业病危害调查[J]. 职业与健康. 2012.
63. 王超英; 汤俊豪; 陈晓琴. 广西某教学口腔医院职业病危害因素调查[J]. 职业与健康. 2008.
64. 王庆荣; 王艳华; 刘宗伟; 邱玉刚; 纪乾鹏; 王婷; 张秀川; 段化伟. 应用 2 种风险评价方法评估某胶合板制造企业甲醛危害风险级别[J]. 中国职业医学. 2017.
65. 袁伟明; 冷朋波; 周莉芳; 邹华; 张美辨. 应用国外两种风险模型评估职业危害的对比研究[J]. 环境与职业医学. 2015.
66. 周志洋; 苏世标; 曾运良. 应用综合指数法评估某制药厂职业健康风险[J]. 中国卫生工程学. 2021.
67. 王维; 冯学庆; 李巧玲. 接触甲醛的职业危害及对嗅觉功能的影响[J]. 中国公共卫生. 1999.
68. 王利军. 控制病理科空气中有毒气体导致的职业风险[J]. 当代医学. 2019.
69. 李晓茹. 攀枝花市某小型彩印厂改造工程职业病危害控制效果评价[J]. 职业与健康. 2014.
70. 轩之英; 滕文革; 李冬梅; 麻小圆. 昌吉州某化工厂甲醛和乌洛托品生产项目职业病危害预评价[J]. 职业与健康. 2015.
71. 霍学义; 杨军红. 木材加工厂职业卫生现状调查及治理对策初探[J]. 微量元素与健康研究. .
72. 罗森林. 木质家具企业职业病危害现状评价[J]. 现代职业安全. 2018.
73. 任鸿; 徐秋凉; 李飞; 王鹏. 木质家具制造企业职业病危害化学因素调查及防治对策[J]. 安全. 2019.
74. 谢禾; 李克勇; 迟美娜; 朱彩菊. 木质家具生产企业职业病危害现状调研[J]. 职业卫生与应急救援. 2012.
75. 常君瑞; 徐东群; 董小艳; 王秦; 唐志刚; 王桂芳; 徐春雨. 板材市场从业人员血清脂质过氧化指标的观察[J]. 环境与健康杂志. 2008.
76. 罗东; 王世松; 彭中全; 谢勇; 钟媛. 某 1,4-丁二醇项目职业病危害控制效果评价[J]. 中国卫生工程学. 2017.
77. 毛一扬; 李小琴; 蔡翔. 某 LED 生产企业空调作业环境有毒物质及空气质量调查分析[J]. 中国卫生工程学. 2018.
78. 张勇; 郭雅蓉; 徐良军. 某乘用车生产基地职业病危害因素的识别与控制对策[J]. 疾病预防控制通报. 2014.
79. 江鑫; 黄同林; 黄宏卿; 张雅慧. 某人造防火板生产企业职业病危害分析与防护措施[J]. 现代预防医学. 2015.

80. 李华亮; 李丽; 熊德甫; 樊晓鹏; 陈敏; 邓倩. 某企业办公室内空气质量及其健康风险评估[J]. Journal of Environmental & Occupational Medicine. 2015.
81. 刘四海, 张辉； 李峰； 杨叔乐； 江建梅. 某企业烙画作业场所甲醛污染的调查分析[J]. 医学动物防制. 2005.
82. 潘健; 付必惠; 何翰成. 某企业玻璃钢复合材料项目职业病危害因素控制效果评价[J]. 应用预防医学. 2014.
83. 付必惠; 潘健; 黄宏辉. 某企业球类生产线职业病危害控制效果评价分析[J]. 实用预防医学. 2012.
84. 王宏峰; 王福祥; 陈彬; 杜再江; 吴爱军; 孟凡艳. 某企业甲醛生产项目职业病危害控制效果评价[J]. 中国卫生工程学. 2010.
85. 陈清洪; 郭华南; 陈煜; 谢德兴; 王晓峰. 某公司年产 5 万吨二甲醚建设项目职业病危害控制效果评价[J]. 中国卫生工程学. 2010.
86. 薛冬梅. 某刨花板厂职业病危害及关键控制点分析[J]. 中国卫生工程学. 2016.
87. 陈坚; 赵远; 马炜钰; 钟坤鹏; 冯玉超; 黄海文; 刘移民. 某制药企业固体制剂技术改造项目职业病危害关键控制点及防控措施分析[J]. 职业卫生与应急救援. 2017.
88. 孙淑君; 穆瑞东. 某制衣厂职业病危害现状调查与评价[J]. 中国新技术新产品. 2015.
89. 陈晓晓; 孙芳; 曹关龄; 石斌; 王怀记; 陈文革. 某办公楼室内环境质量分析[J]. CHINESE JOURNAL OF PUBLIC HEALTH ENGINEERING. 2009.
90. 王宏峰; 陈彬; 郭晓诗; 李兆辉; 杨平; 付长峰. 某包装物厂职业病危害控制效果评价[J]. 中国卫生工程学. 2009.
91. 柳春; 曾锐志; 秦小洁; 王星力; 王维平; 王晓捷. 某化工企业化学危害呼吸防护现状调查[J]. 中国卫生工程学. 2013.
92. 陈凤琼; 冉瑞红; 杨迪. 某化工厂纺丝车间职业病危害现状分析[J]. 中国卫生工程学. 2017.
93. 戴雪松; 周桂侠; 李焕焕; 赵杰. 某化工扩链剂和可湿粉生产线职业病危害及关键控制点分析[J]. 职业卫生与应急救援. 2014.
94. 周鉴. 某医疗机构病理科冰冻切片室职业病危害调查[J]. 中国职业医学. 2019.
95. 刘磊; 陈栋; 程婷婷; 唐昆; 蔡秀秀; 薛腾飞; 李鹏飞; 姜正好; 李开春. 某医疗机构病理科甲醛和二甲苯职业健康风险评估[J]. 实用预防医学. 2020.
96. 冯云; 李红英; 袁月琳. 某医院改建项目职业病危害预评价[J]. 中国卫生工程学. 2008.
97. 张熔熔; 唐威; 蔡颖; 孙屏. 某医院病理科空气污染现状及解决方法初探[J]. JOURNAL OF ENVIRONMENTAL & OCCUPATIONAL MEDICINE. 2008.
98. 刘永泉, 艾林芳； 张陆兵； 李建昌； 汤昌海. 某医院病理科职业病危害因素现状调查[J]. 中华劳动卫生职业病杂志. 2021.
99. 黄灵; 刘新荣; 沈骏. 某原料药生产企业职业病危害评价要点[J]. 现代预防医学. 2010.

100. 张玉彬; 王法弟; 稽留中; 张国英; 姚永平; 许卫兰; 沈光祖; 夏昭林. 某县木材装饰贴面加工业冬季作业环境调查[J]. 工业卫生与职业病. 2008.
101. 黎长蕊; 翟秀芳. 某商场环境污染状况调查研究[J]. OCCUPATIONAL HEALTH AND EMERGENCY RESCUE. 2003.
102. 周君; 黄德寅. 某大型化工厂甲醛-聚甲醛装置职业病危害因素关键控制点分析[J]. 职业卫生与应急救援. 2013.
103. 叶宏; 门晓棠; 关晶萍; 许文博; 宋素合; 黄力维. 某大型化肥企业尿素包装贮运系统职业病危害及关键控制点分析[J]. 中国工业医学杂志. 2014.
104. 黎海红; 江世强; 聂传丽. 某大型汽车制造企业工人接触空气中化学物质情况调查[J]. 中国职业医学. 2012.
105. 郑利纬; 杜向阳. 某家具制造厂新建项目职业病危害控制效果评价[J]. 职业与健康. 2009.
106. 李清虹; 刘胜勇; 程志勇; 杨风艳. 某家具厂职业病危害因素调查[J]. 工业卫生与职业病. 2011.
107. 黄琼; 柯宗枝; 商群; 高忠; 李晓莉. 某市人造板生产企业职业卫生现状调查[J]. 海峡预防医学杂志. 2015.
108. 毕章华; 张恒东; 许玲珍. 某市木制家具制造厂职业病危害干预效果初评估[J]. 职业卫生与应急救援. 2013.
109. 沈月华; 杜洪凤; 陈俊华. 某建设项目职业病危害控制效果评价[J]. 现代预防医学. 2006.
110. 张宏; 厚丽华; 王艳杰; 王晶. 某微型汽车橡塑件建设项目控制效果评价[J]. 职业与健康. 2006.
111. 朱江丽; 万立力; 王强强; 胡志. 某扩建项目职业病危害预评价[J]. 现代矿业. 2017.
112. 李克勇; 黄沪涛; 赵乾魁; 孙原; 刘武忠. 某拉链制造企业甲醛职业接触致癌风险评估[J]. 职业卫生与应急救援. 2017.
113. 孙守锋; 赵国坡; 刘文凯; 李航. 某新建浸胶帘子布建设项目职业病危害因素调查与分析[J]. 青海科技. 2021.
114. 刘前; 齐新周; 谭春艳. 某新建浸胶帘子布建设项目职业病危害预评价[J]. 中国工业医学杂志. 2012.
115. 王聪伟; 方绍峰. 某无碳复写纸专用树脂显色剂工程项目职业卫生学调查与评价[J]. 职业卫生与应急救援. 2006.
116. 郭防. 某木业公司职业病危害识别与关键控制点分析[J]. 医学信息(中旬刊). 2011.
117. 刘昌伟; 贺法宪; 董庶军; 王洪涛; 代振华. 某木业加工厂职业病危害现状调查分析[J]. 中国城乡企业卫生. 2013.
118. 张诗军. 某木制家具制造企业职业病危害因素的检测与分析[J]. 职业与健康. 2012.
119. 张弦; 李明. 某木制板厂职业病危害因素识别及防控对策[J]. 职业与健康. 2010.

120. 徐兰. 某木质家具生产项目职业病危害控制效果评价[J]. China Science & Technology Panorama Magazine. 2018.
121. 卿文静; 林孟端; 林海端; 许志恒. 某机械五金有限公司职业病危害控制效果评价[J]. 职业与健康. 2016.
122. 吴诗华; 曾文锋; 刘丽芬; 刘移民. 某板业有限公司职业病危害控制效果评价[J]. 职业与健康. 2013.
123. 江为科. 某毛绒玩具厂职业病危害控制效果评价[J]. 全科口腔医学电子杂志. 2018.
124. 陶玲; 卫海燕; 张士怀; 张海东; 陈术坤; 张普. 某氨基模塑料生产企业职业危害现况分析[J]. 中国工业医学杂志. 2021.
125. 郭少红; 张颖; 詹玉贞. 某污水处理厂职业病危害控制效果评价[J]. 职业卫生与应急救援. 2016.
126. 邱劲松; 彭言群; 龚禧; 曹芳; 李躲; 贺性鹏. 某汽车刹车片作业岗位职业病危害程度分级评价[J]. 中国工业医学杂志. 2013.
127. 张鸿; 赵淑岚. 某汽车发动机有限公司职业卫生现场调查与评价[J]. 职业与健康. 2012.
128. 王志文; 胡爽; 刘凯; 沈颖惠. 某汽车部件厂职业病危害现状评价[J]. 中国卫生产业. 2019.
129. 王致; 张海; 梁嘉斌; 廖阳; 张燕; 刘移民. 某汽车隔音件技术改造项目职业病危害控制效果评价[J]. 中国卫生工程学. 2011.
130. 杜影; 邵小翠; 安玉. 某汽车零部件铸业公司扩建项目职业病危害现状评价[J]. 职业与健康. 2014.
131. 潘贵和; 纪晓辉; 张义田; 周红梅. 某油田原油脱水剂成分及其职业接触检测[J]. 工业卫生与职业病. 2011.
132. 李承浩; 孙建娅; 韩凯. 某玛钢加工公司职业病危害控制效果分析评价[J]. 中国药物与临床. 2021.
133. 杜春玲; 董磊; 燕东剑. 某玻璃纤维湿法薄毡生产线职业病危害防护措施及关键控制点分析[J]. 中国卫生工程学. 2016.
134. 刘国中. 某甲醛厂职业病危害因素调查分析[J]. 中国伤残医学. 2013.
135. 王世松; 罗东; 彭中全; 谢勇; 钟媛. 某甲醛项目职业病危害控制效果评价[J]. 中国卫生工程学. 2015.
136. 金鹏. 某电子企业 180 万 m2/a 电路板生产线项目职业病危害控制效果评价[J]. 中国科技期刊数据库 科研. 2015.
137. 张耘; 肖鸣云. 某电子企业纸基覆铜线路板项目职业病危害的控制效果评价[J]. 职业与健康. 2011.
138. 费小进; 童智敏; 杜成; 孟谦谦. 某电路板厂职业病危害控制效果评价[J]. 职业与健康. 2011.
139. 陈凤琼; 黄进; 邹碧海. 某皮鞋生产项目职业病危害控制效果评价[J]. 中国工业医学杂志. 2012.
140. 冯柳羽; 陈达希; 翁隽. 某矿棉板材制造企业职业病危害现状评价[J]. 职业与健康. 2013.

141. 程茂定; 屠鹃; 张金龙. 某红木家具制造企业职业病危害因素调查与关键控制点分析[J]. 中国卫生工程学. 2019.
142. 卢建国; 胡雪松; 谢永宽. 某纤维板厂甲醛危害及其关键控制点分析[J]. 职业卫生与应急救援. 2012.
143. 刘素芹; 于瑞广. 某草柳木制品厂职业病危害因素评价[J]. 职业与健康. 2007.
144. 党庆德; 王坤; 胡永超; 魏来. 某草甘膦生产建设项目职业病危害控制效果评价[J]. 中国卫生工程学. 2011.
145. 常艾民; 吴群煌; 王艳彬. 某车床铸造建设项目职业病危害控制效果评价[J]. 中国工业医学杂志. 2016.
146. 聂云峰; 彭仁和; 董吉良; 何卫红; 胡建安. 某造船厂职业病危害因素调查[J]. 实用预防医学. 2011.
147. 李长松; 刘洪强; 单永乐; 吴洪涛; 张放. 某采油厂修井作业职业病危害调查与评价[J]. 中国辐射卫生. 2018.
148. 黄琼; 潘绥; 张晓峰; 陈珊. 某铸造企业职业危害及作业分级调查[J]. 海峡预防医学杂志. 2019.
149. 刘晓秋, 高群；郑晶；任刚. 某防盗门业建设项目职业病危害控制效果评价[J]. 中外医学研究. 2009.
150. 朱海洲; 杨晓发. 某阻燃装饰板工程项目职业卫生学评价[J]. 中国工业医学杂志. 2005. 1002-221X
151. 卢建国; 胡雪松; 谢永宽. 某高密度纤维板厂甲醛危害及其关键控制点分析[J]. 职业卫生与病伤. 2012.
152. 卢建国, 温伟华；周惠胡. 某高密纤维板厂甲醛作业工人健康状况的调查分析[J]. 国际医药卫生导报. 2011.
153. 温伟华; 何寿国; 许振国. 某高密纤维板生产企业甲醛职业病的控制效果分析[J]. 广东医学院学报. 2013.
154. 万国林; 甘为民; 周银平; 谭向文; 李继国; 吴红娥. 档案库房职业危害对人体健康的影响[J]. 中国工业医学杂志. 2000.
155. 宋向荣; 张爱华; 蒙瑞波; 蔡婷峰. 毒性病理实验室职业病危害因素识别与检测[J]. 职业卫生与应急救援. 2019.
156. 顾凯风; 吴文; 宋伟. 江苏省某公司高档家具生产线升级技术改造及扩建项目职业病危害预评价[J]. 职业与健康. 2019.
157. 舒丽萍; 王强; 廖云华; 沈卫星. 污水处理厂职业病危害因素检测与分析[J]. 中国卫生检验杂志. 2009.
158. 秦彩明; 柏中波; 孔庆宇; 陈杵序; 邹积慧. 沈阳市纸质印刷企业职业病危害因素的调查[J]. 职业与健康. 2014.

159. 侯广庆; 平庆玲; 周晓蓉. 油田三次采油技术开采的职业病危害因素调查[J]. 职业卫生与应急救援. 2014.
160. 轩志东. 油田配酸、酸化及压裂作业的劳动卫生学调查及初步比较研究[J]. 河南预防医学杂志. 2001.
161. 邵洪. 油田配酸工人职业性损害的调查[J]. 工业卫生与职业病. 1989.
162. 孔凡玲; 李仁波; 高衍新; 王德军; 隋少峰. 济南市 6 家综合医院病理科室内空气甲醛浓度及变化特征[J]. 环境与职业医学. 2015.
163. 丁雯; 夏猛; 侯学文; 姜程; 李宁. 淄博市 10 家医院病理科环境中甲醛、二甲苯危害的调查[J]. Chinese Journal of Industrial Medicine. 2015.
164. 蒋立新; 郑晓钧; 李汉锋; 潘瑞胤; 杨梅. 深圳市福田区电子市场挥发性化学品污染状况的调查[J]. 职业与健康. 2014.
165. 蒋立新; 郑晓均; 李汉锋; 潘瑞胤; 杨梅. 深圳市福田区电子市场职业卫生现况调查[J]. 预防医学情报杂志. 2010.
166. 颜复宝. 湖南绥宁县 5 个企业甲醛作业的职业危害调查[J]. 职业与健康. 2005.
167. 赵阳辉; 黄日生; 李春萍; 冯忠海. 湛江市某船厂作业场所职业卫生现况调查[J]. 职业与健康. 2013.
168. 颜彩虹; 易继湖. 甲醛作业人员的健康状况:以 3 家密度板制造企业为例[J]. 环境与职业医学. 2015.
169. 李芝兰; 连素琴; 蔡辉民; 张峰; 裴泓波. 甲醛对人体职业危害的调查[J]. 中国职业医学. 1999.
170. 金复生; 朱瑞娟. 甲醛对从业工人外周血淋巴细胞的遗传学效应[J]. 中国劳动卫生职业病杂志. 1992. 1001-9391
171. 童智敏; 施健; 杜成; 费小进. 甲醛对作业工人免疫系统的损伤作用[J]. 环境与职业医学. 2010.
172. 周烨, 王维； 王秋萍. 甲醛对作业工人呼吸系统及肺功能的影响[J]. 中国工业医学杂志. 2000.
173. 袁小兰; 袁小燕; 杨恒. 甲醛对心血管系统的影响[J]. JOURNAL OF MATHEMATICAL MEDICINE. 2011.
174. 施健; 朱士新; 童智敏; 孙东晓; 杨红; 姜荣明; 孔璐; 赵进顺. 甲醛对职业接触工人健康效应的流行病学调查[J]. 中国职业医学. 2006.
175. 张瑞德; 蒋学之. 甲醛接触工人的工作有关疾病[J]. 职业卫生与应急救援. 1990.
176. 郭金峰; 刘锦红; 稽春妹; 王伟; 陈震炎; 金凤娟. 甲醛的职业危害[J]. 中华劳动卫生职业病杂志. 1988. 1001-9391
177. 俞绍武. 甲醛职业危害的调查[J]. 化工劳动保护(工业卫生与职业病分册). 1988.
178. 张淼; 夏海娜. 病理科工作环境中的空气污染与控制措施[J]. Journal of Traditional Chinese Medicine Management. 2017.

179. 袁慧敏; 李薇. 病理科工作环境的空气污染与控制措施[J]. Journal of Traditional Chinese Medicine Management. 2015.
180. 王伟. 病理科工作环境的空气污染及其控制对策探析[J]. Health Guide. 2016.
181. 景华; 张放; 李侠. 病理科职业危害因素调查[J]. 卫生软科学. 2017.
182. 唐文娟; 柯宗枝; 魏陈军; 高忠; 王世栋. 纺织印染中小企业主要职业病危害风险综合评估[J]. 海峡预防医学杂志. 2011.
183. 冷朋波; 边国林; 王爱红; 王群利; 张美辨. 美国 EPA 吸入风险模型在木质家具制造企业职业健康风险评估中的应用[J]. 环境与职业医学. 2014.
184. 梁晓军; 嵇心怡; 张宏斌; 胡斌; 徐胜; 赵萍. 美容美发场所主要污染物暴露特征及从业人群健康风险评价[J]. 江苏预防医学. 2019.
185. 张英英. 职业卫生标准 GBZ / T160 · 54-2007 中存在问题及探讨[J]. 中华预防医学杂志. 2012.
186. 翟金霞; 冯丫娟; 方四新; 丁书姝; 张照祥; 王兴华; 杨永坚. 职业性接触甲醛作业人员的神经行为功能变化[J]. 环境与健康杂志. 2010.
187. 施健; 童智敏; 姜荣明. 职业接触甲醛对工人神经行为功能的影响[J]. 环境与职业医学. 2007.
188. 丁璐; 胡凤霞; 姚建华; 杨跃新; 朱宝立. 苏州市某电子设备制造企业职业病危害现状评价[J]. 职业与健康. 2018.
189. 宋仙平; 蔡文妍; 刘炘; 张峰; 朱宝立. 草甘膦生产企业职业病危害调查[J]. 职业卫生与应急救援. 2019.
190. 刘本先; 孙永波; 官洪国. 装修材料挥发有害物质对装修工人血清中 SOD 酶活性及 MDA 含量的影响[J]. 中国卫生工程学. 2011.
191. 姚永平; 张国英; 唐智峰; 王学才; 许卫兰. 装饰面板企业生产车间空气中甲醛检测结果及其影响因素分析[J]. 职业卫生与应急救援. 2010.
192. 索婷婷; 樊立军. 西安市某汽车座椅有限公司整体搬迁改造建设项目职业病危害控制效果评价[J]. 职业与健康. 2020.
193. 张忠群; 李中帅; 任瑞枚; 王淑青; 张华强. 进口涤纶面料所致加工工人职业危害调查[J]. 中国工业医学杂志. 2000.
194. 梁道康; 熊欢; 辜良莉. 重庆市 3 家油漆厂职业病危害检测结果分析[J]. 中国卫生工程学. 2018.
195. 梁道康; 梁利. 重庆市巴南区生产企业发生急性职业中毒的潜在性[J]. 职业与健康. 2008.
196. 陈华磊; 汤娟; 梁道康. 重庆市某中型印染企业职业病危害因素调查及防护建议[J]. 职业与健康. 2014.
197. 曹磊; 冉瑞红. 重庆市某木制家具制造企业职业病危害因素及其关键控制点分析[J]. 职业与健康. 2020.
198. 陈凤琼; 冉瑞红. 重庆市某汽车内饰系统生产企业职业病危害现状[J]. 职业与健康. 2017.

199.　刘沈; 陈华磊; 汤娟; 梁道康. 重庆市某漆业制造公司职业病危害现状评价[J]. 职业与健康. 2014.
200.　汪运; 邱建平; 彭中全. 重庆市某甲醚化三聚氰胺树脂生产企业职业危害情况调查[J]. 职业与健康. 2018.
201.　曹磊; 陈凤琼. 重庆市某钻井材料生产企业职业病危害因素及关键控制点[J]. 职业与健康. 2019.
202.　李小平; 杨迪; 邓华欣; 邵成. 重庆某大型微型车项目职业病危害控制效果评价[J]. 职业与健康. 2016.
203.　王雷. 铁路隧道维修作业急性职业性危害因素调查[J]. 中华劳动卫生职业病杂志. 2014.
204.　汪运; 汪志鹏; 彭中全; 谢勇; 张立; 田平. 银法和铁钼法甲醛生产工艺外操岗位甲醛暴露职业健康风险比较[J]. 职业卫生与应急救援. 2021.
205.　万学文，熊世洲，刘继纯，梅勇，姚道华. 铸造业树脂砂职业危害及实验研究[J]. 预防医学情报杂志. 1994.
206.　孟传三, 张敏；祁成；陈卫红；鲁洋；杜燮祎；李文捷. 铸造作业职业性有害因素及其特点的再分析[J]. 中华劳动卫生职业病杂志. 2010.
207.　陈云. 预防纺织品中游离甲醛的职业性接触[J]. 职业与健康. 1987.
208.　张陆兵; 田月; 陈绍寿; 熊金勇; 刘永泉. 高铁机车驾驶员职业健康和心理健康状况分析[J]. 中国职业医学. 2019.
209.　屠悦健; 李国春; 孔海宏. 黄浦区 3 类公共场所甲醛污染及接触人群症状调查[J]. Shanghai Journal of Preventive Medicine. 2011.
210.　金佰明. 齐齐哈尔市某家居石材加工作坊甲醛职业危害评价[J]. 齐齐哈尔医学院学报. 2012.
211.　梁永锡,刘可平,陈浩,等. 广东省某市 202 家电子企业职业病危害因素现状调查 [J]. 职业卫生与应急救援, 2024, 42 (01): 45-48+62. DOI:10.16369/j.oher.issn.1007-1326.2024.01.009.
212.　杨庆,蒋显聪,刘秀红,等. A/O 工艺污水处理厂醛酮化合物释放特征及风险评价 [J]. 北京工业大学学报, 2023, 49 (12): 1338-1347.
213.　史蓉婕,黄振翔,王礼法,等. 2016—2021 年深圳市罗湖区美容美发场所健康危害因素分析 [J]. 环境卫生学杂志, 2023, 13 (10): 752-756. DOI:10.13421/j.cnki.hjwsxzz.2023.10.006.
214.　高循洲,单炳丹,王慈媛,等. 应用 EPA 吸入模型评估木工作业中甲醛的职业健康风险水平 [J]. 工业卫生与职业病, 2023, 49 (04): 334-337. DOI:10.13692/j.cnki.gywsyzyb.2023.04.011.
215.　张宏,张海东. 某大型木制家具生产企业职业病危害控制效果评价 [J]. 中国工业医学杂志, 2023, 36 (03): 263-266. DOI:10.13631/j.cnki.zggyyx.2023.03.021.
216.　吴一鸣,王顺恺,江建勋,等. 十堰市理发及美容场所室内空气质量对从业人员健康的影响 [J]. 环境卫生学杂志, 2023, 13 (05): 378-385. DOI:10.13421/j.cnki.hjwsxzz.2023.05.012.

217.	沈磊,张海云,李晓红,等. 兰州市某生物医药制品生产项目职业病危害控制效果评价 [J]. 职业与健康, 2023, 39 (16): 2179-2183. DOI:10.13329/j.cnki.zyyjk.20230426.004.
218.	汪凤娇,田海霞,侯文胜,等. 廊坊市人造板生产企业甲醛接触岗位职业健康风险评估 [J]. 中国工业医学杂志, 2023, 36 (02): 178-181. DOI:10.13631/j.cnki.zggyyx.2023.02.024.
219.	高衍新,张晓,江媛媛,等. 聊城市宾馆客房气态污染物暴露特征及对客房从业人员健康风险评估 [J]. 环境卫生学杂志, 2023, 13 (02): 117-122. DOI:10.13421/j.cnki.hjwsxzz.2023.02.008.
220.	张莹, 马宇熙, 张勇等. 2021-2022 年西北某城市地铁环境空气质量调查研究[J]. 环境与职业医学, 2023, 40(11): 1290-1296. DOI: 10.11836/JEOM23034
221.	张莉萍,倪骏,郑毅鸣等.上海市大型展会部分室内空气污染物分布特征与健康风险评价[J].环境与职业医学,2021,38(05):489-493.DOI:10.13213/j.cnki.jeom.2021.20381.
222.	杜英林, 张晓, 周汝彬等. 聊城市理发美容场所空气中常见化学污染物暴露特征及从业人员健康风险评估[J]. 环境与职业医学, 2023, 40(3): 342-348. DOI: 10.11836/JEOM22241
223.	Li W, Zhang M, Zhao T. [A study on the control of air toxic chemicals in workplace for bakelite manufacturing and casting]. Zhonghua Yu Fang Yi Xue Za Zhi. 1996 Nov;30(6):357-9. Chinese. PMID: 9388913.
224.	Su M, Sun R, Zhang X, Wang S, Zhang P, Yuan Z, Liu C, Wang Q. Assessment of the inhalation risks associated with working in printing rooms: a study on the staff of eight printing rooms in Beijing, China. Environ Sci Pollut Res Int. 2018 Jun;25(17):17137-17143. doi: 10.1007/s11356-018-1802-z. Epub 2018 Apr 11. PMID: 29644615.
225.	Feng Y, Mu C, Zhai J, Li J, Zou T. Characteristics and personal exposures of carbonyl compounds in the subway stations and in-subway trains of Shanghai, China. J Hazard Mater. 2010 Nov 15;183(1-3):574-82. doi: 10.1016/j.jhazmat.2010.07.062. Epub 2010 Jul 21. PMID: 20692096.
226.	Dai W, Zhong H, Li L, Cao J, Huang Y, Shen M, Wang L, Dong J, Tie X, Ho SSH, Ho KF. Characterization and health risk assessment of airborne pollutants in commercial restaurants in northwestern China: Under a low ventilation condition in wintertime. Sci Total Environ. 2018 Aug 15;633:308-316. doi: 10.1016/j.scitotenv.2018.03.124. Epub 2018 Mar 23. PMID: 29574375.
227.	Ye X, Yan W, Xie H, Zhao M, Ying C. Cytogenetic analysis of nasal mucosa cells and lymphocytes from high-level long-term formaldehyde exposed workers and low-level short-term exposed waiters. Mutat Res. 2005 Dec 7;588(1):22-7. doi: 10.1016/j.mrgentox.2005.08.005. Epub 2005 Oct 28. PMID: 16257574.
228.	Wang K, Wang TW, Xu J, Zhu Y, Jian L, Au W, Xia ZL. Determination of benchmark dose based on adduct and micronucleus formations in formaldehyde-exposed workers. Int J Hyg Environ Health. 2019 Jun;222(5):738-743. doi: 10.1016/j.ijheh.2019.05.008. Epub 2019 May 28. PMID: 31147150.
229.	Jia X, Jia Q, Zhang Z, Gao W, Zhang X, Niu Y, Meng T, Feng B, Duan H, Ye M, Dai Y, Jia Z, Zheng Y. Effects of formaldehyde on lymphocyte subsets and cytokines in the peripheral blood of exposed workers. PLoS One. 2014 Aug 26;9(8):e104069. doi: 10.1371/journal.pone.0104069. PMID: 25157974; PMCID: PMC4144836.
230.	Lu YY, Lin Y, Zhang H, Ding D, Sun X, Huang Q, Lin L, Chen YJ, Chi YL, Dong S. Evaluation of Volatile Organic Compounds and Carbonyl Compounds Present in the Cabins of Newly Produced, Medium- and Large-Size Coaches in China. Int J Environ Res Public Health. 2016 Jun 15;13(6):596. doi: 10.3390/ijerph13060596. PMID: 27314375; PMCID: PMC4924053.

231. Ai LF, Zhang LB, Li JC, Tang CH, Liu YQ. [Formaldehyde and xylene levels and protective effects in the pathology department of a hospital]. Zhonghua Lao Dong Wei Sheng Zhi Ye Bing Za Zhi. 2021 Jan 20;39(1):64-65. Chinese. doi: 10.3760/cma.j.cn121094-20200415-00195. PMID: 33535348.
232. Liang X, Zhang J, Song W, Wang K, Zhang B. Formaldehyde Exposure in Indoor Air From Public Places and Its Associated Health Risks in Kunshan City, China. Asia Pac J Public Health. 2018 Sep;30(6):551-560. doi: 10.1177/1010539518800348. Epub 2018 Sep 15. PMID: 30221985.
233. Mui K, Wong L, Hui P, Chan W. Formaldehyde exposure risk in air-conditioned offices of Hong Kong. Building Services Engineering Research and Technology. 2009;30(4):279-286. doi:10.1177/0143624409339613
234. Ho SS, Ip HS, Ho KF, Ng LP, Chan CS, Dai WT, Cao JJ. Hazardous airborne carbonyls emissions in industrial workplaces in China. J Air Waste Manag Assoc. 2013 Jul;63(7):864-77. doi: 10.1080/10962247.2013.797519. PMID: 23926855.
235. Gong Y, Wei Y, Cheng J, Jiang T, Chen L, Xu B. Health risk assessment and personal exposure to Volatile Organic Compounds (VOCs) in metro carriages - A case study in Shanghai, China. Sci Total Environ. 2017 Jan 1;574:1432-1438. doi: 10.1016/j.scitotenv.2016.08.072. Epub 2016 Aug 14. PMID: 27535570.
236. Qin D, Guo B, Zhou J, Cheng H, Chen X. Indoor air formaldehyde (HCHO) pollution of urban coach cabins. Sci Rep. 2020 Jan 15;10(1):332. doi: 10.1038/s41598-019-57263-4. PMID: 31941990; PMCID: PMC6962397.
237. Yin Y, He J, Pei J, Yang X, Sun Y, Cui X, Lin CH, Wei D, Chen Q. Influencing factors of carbonyl compounds and other VOCs in commercial airliner cabins: On-board investigation of 56 flights. Indoor Air. 2021 Nov;31(6):2084-2098. doi: 10.1111/ina.12903. Epub 2021 Jul 9. PMID: 34240486.
238. Hong, W., Meng, M., Xie, J., Gao, D., Zeng, Y., Ai, H., Chen, C., Huang, S. and Zhou, Z. (2017). Investigation of the Pollution Level and Affecting Factors of Formaldehyde in Typical Public Places in Guangxi, China. *Aerosol Air Qual. Res.* 17: 2816-2828. https://doi.org/10.4209/aaqr.2017.08.0272
239. Lü, H., Tian, J.J., Cai, Q.Y., Wen, S., Liu, Y. and Li, N. (2016). Levels and Health Risk of Carbonyl Compounds in Air of the Library in Guangzhou, South China. *Aerosol Air Qual. Res.* 16: 1234-1243. https://doi.org/10.4209/aaqr.2015.09.0533
240. Ying CJ, Yan WS, Zhao MY, Ye XL, Xie H, Yin SY, Zhu XS. Micronuclei in nasal mucosa, oral mucosa and lymphocytes in students exposed to formaldehyde vapor in anatomy class. Biomed Environ Sci. 1997 Dec;10(4):451-5. PMID: 9448927.
241. Lin D, Guo Y, Yi J, Kuang D, Li X, Deng H, Huang K, Guan L, He Y, Zhang X, Hu D, Zhang Z, Zheng H, Zhang X, McHale CM, Zhang L, Wu T. Occupational exposure to formaldehyde and genetic damage in the peripheral blood lymphocytes of plywood workers. J Occup Health. 2013;55(4):284-91. doi: 10.1539/joh.12-0288-oa. Epub 2013 May 2. PMID: 23648472.
242. Zhang L, Tang X, Rothman N, Vermeulen R, Ji Z, Shen M, Qiu C, Guo W, Liu S, Reiss B, Freeman LB, Ge Y, Hubbard AE, Hua M, Blair A, Galvan N, Ruan X, Alter BP, Xin KX, Li S, Moore LE, Kim S, Xie Y, Hayes RB, Azuma M, Hauptmann M, Xiong J, Stewart P, Li L, Rappaport SM, Huang H, Fraumeni JF Jr, Smith MT, Lan Q. Occupational exposure to formaldehyde, hematotoxicity, and leukemia-specific chromosome changes in cultured myeloid progenitor cells. Cancer Epidemiol Biomarkers Prev. 2010 Jan;19(1):80-8. doi: 10.1158/1055-9965.EPI-09-0762. PMID: 20056626; PMCID: PMC2974570.
243. Xu H, Zhang Q, Song N, Guo M, Zhang S, Ji G, Shi L. Personal exposure and health risk assessment of carbonyls in family cars and public transports-a comparative study in Nanjing, China. Environ Sci Pollut Res Int. 2017 Nov;24(33):26111-26119. doi: 10.1007/s11356-017-0150-8. Epub 2017 Sep 24. PMID: 28944438.

244. Mui KW, Wong LT, Hui PS. Policy Influence of Formaldehyde Exposure Risk in Air-conditioned Office Environment. Indoor and Built Environment. 2008;17(5):449-454. doi:10.1177/1420326X08096110
245. Wang H, Feng D, He Y, Jin X, Fu S. Comprehensive interventions to reduce occupational hazards among medical staff in the pathology department of five primary hospitals. BMC Public Health. 2023;23(1):2136. Published 2023 Oct 31. doi:10.1186/s12889-023-16948-2
246. Wong LT, Mui KW, Hui PS. Screening for Indoor air Quality of Air-Conditioned Offices. Indoor and Built Environment. 2007;16(5):438-443. doi:10.1177/1420326X07082159

Supplementary Material 3: Standards of sampling, determination, and Occupational Exposure Limits for Formaldehyde

1. Sampling

Earlier in China, there were no standards for sampling until the (***WS1-1996) Workplace air-Determination of toxic substances-Sampling strategy*** was published in 1996. According to this document, absorption tubes with 5 ml absorption liquid were used to sample 15 minutes, representing the average concentration of 15 minutes. When sampling time is less than 5 minutes (depending on the task and sample methods), one should have several samples with a sum of sampling time equal to 15 minutes. By 2004, the (***GBZ 159-2004) Specifications of air sampling for hazardous substances monitoring in the workplace*** finalized sampling for chemicals using maximum allowed concentration (MAC) as occupational exposure level (OEL) such as formaldehyde. Short-term sampling should be used to collect air samples for 15 minutes at the representative and most exposed area in the workers' breath zone.

Recorded air-quality inspection assessments in various settings also used an absorption tube with 5 ml absorption liquid to collect 10 L air samples at a flow rate of 0.5 L/min. The related standards included: ***(GB/T 18204.26-2000) Methods for determination of formaldehyde in air of public places, (GB/T 18204.2-2010) Examination methods for public places-Part 2: Chemical pollutants, (GB/T 18204.2-2014) Examination methods for public places-Part 2: Chemical pollutants.***

2. Sample analysis

Accordingly, Gas Chromatography was used for sample analysis which could refer to the ***(GB/T16057-1995) Workplace air--Determination of formaldehyde--3-Methyl-2-benzothiazolinone hydrazone hydrochloride (MBTH) spectrophotometric method; (WS/T150-1999) Workplace air. Determination of formaldehyde. Polarographic method; (GBZ/T160.54-2004) Methods for determination of aliphatic aldehydes in the air of work place; (GBZ/T160.54-2007) Determination of aliphatic aldehydes in the air of workplace; (GBZ/T300.99-2017) Determination of toxic substances in workplace air Part 99: Formaldehyde, acetaldehyde and butyraldehyde.***

3. Occupational Exposure Limits (OEL)

In 1979, the Ministry of Health (MOH) established a MAC of 3 mg/m^3 (MOH, 1979) as the only OEL for formaldehyde in the Chinese National Standard: **(TJ 36-79)** ***Hygienic standards for the design of industrial premises*****.** In 2002, that value was significantly reduced to 0.5 mg/m^3 in the ***(GBZ1-2002) Hygienic standards for the design of industrial premises***, and remains the occupational standard today ***in the (GBZ2.1-2007) Occupational exposure limits for hazardous agents in the workplace***.
